# The effect of high fat diet and metformin treatment on liver lipids accumulation and their impact on insulin action

**DOI:** 10.1038/s41598-018-25397-6

**Published:** 2018-05-08

**Authors:** Piotr Zabielski, Hady Razak Hady, Marta Chacinska, Kamila Roszczyc, Jan Gorski, Agnieszka U. Blachnio-Zabielska

**Affiliations:** 1 Department of Medical Biology, Medical University of Bialystok, Bialystok, Poland; 2 Department of Physiology, Medical University of Bialystok, Bialystok, Poland; 3 1st Department of General Surgery and Endocrinology, Medical University Bialystok, Bialystok, Poland; 4 Department of Hygiene, Epidemiology and Metabolic Disorders, Medical University of Bialystok, Bialystok, Poland; 5 Department of Basic Sciences, Faculty of Health Sciences, Lomza State University of Applied Sciences, Lomza, Poland

## Abstract

We sought to determine whether metformin treatment reverses a high-fat diet (HFD)-induced hepatic insulin resistance (IRes) and to identify lipid intermediates involved in induction of liver IRes. The experiments were conducted on male Wistar rats divided into three groups: 1. Control, 2. fed HFD and 3. fed HFD and treated with metformin. The animals were infused with a [U-^13^C]palmitate to measure fractional lipid synthesis rate. This allowed for the calculation of fractional synthesis rate of signaling lipids (FSR) through the estimation of their isotopic enrichment. Liver ceramide (Cer), diacylglycerol (DAG) and acyl-carnitine concentration and enrichment were analyzed by LC/MS/MS. The content of proteins involved in lipid metabolism and insulin signaling were analyzed by Western Blot. HFD treatment increased the content and FSR of DAG and Cer in the liver which was accompanied by systemic insulin resistance and inhibition of hepatic insulin signaling pathway under insulin stimulation. Metformin treatment ameliorated systemic insulin resistance and augmented the hepatic insulin signaling cascade. It reduced both the concentration and FSR of Cer, DAG, and increased acyl-carnitine content and the expression of mitochondrial markers. We postulate, that in liver, the insulin sensitizing effect of metformin depends on augmentation of mitochondrial β-oxidation, which protects from hepatic accumulation of both the Cer and DAG and preserves insulin sensitivity under HFD consumption. Moreover, we showed that hepatic content of Cer and DAG corresponds with their respective FSR.

## Introduction

The liver, in addition to adipose tissue and skeletal muscle, is a key player in regulation of glucose and lipid metabolism. Obesity and high fat diet (HFD) are associated with intracellular lipid accumulation. It has been found that even short term HFD feeding in rodents results in hepatic fat accumulation and insulin resistance (IRes)^1^. In the physiological state, the main role of insulin in liver is the inhibition of gluconeogenesis in the presence of high plasma glucose level. In the state of insulin resistance, glucose output from liver increases due to hepatic IRes. The main factors that contribute to this state are as follows: increased rate of hepatic gluconeogenesis and defects in insulin-stimulated hepatic glycogen synthesis. Metformin, the widely used insulin-sensitizing drug, leads to reduction of hepatic glucose production^2,3^, but the mechanism by which metformin inhibits hepatic gluconeogenesis still remains unknown.

It is commonly accepted that hepatic lipid accumulation is associated with the induction of IRes, but despite decades of intense investigation, pathogenesis of IRes still remains incompletely understood. The excess of plasma free fatty acids (FFA) appears to play a significant role in the development of IRes and type 2 diabetes (T2D). In such condition, peripheral tissues, like skeletal muscle and liver, uptake more plasma FA. The visceral adipose tissue is drained by the portal venous system, which has direct connection with the liver. The increased FA disposal stimulates gluconeogenesis in the liver and consequently increases plasma glucose concentration^4,5^. Plasma FFAs enter the cell through diffusion or protein-mediated transport^6^. There are three groups of fatty acids (FA) transporters: fatty acid translocase (FAT/CD36), fatty acid binding protein (FABPpm), and fatty acid transport protein (FATP1–6)^6–8^. The primary FA transporters in hepatocytes are FATP5 and FATP2. After entering the cell, FA are activated by acyl-CoA synthethase (ACSVL1) to form a fatty acyl-CoA (FA-CoA), which then enters the mitochondria for oxidation or are directed to the synthesis of intracellular lipids^9^. LCA-CoAs are transported across the mitochondrial membrane as acyl-carnitines which are synthesized by carnitine palmitoyltransferase 1 (CPT1). Malonyl-CoA, a key intermediate in fatty acids *de novo* synthesis, blocks lipid transport into mitochondria through inhibition of CPT1 and in this way also mitochondrial β-oxidation. Metformin, inhibits acyl-CoA carboxylase (ACC) by activation of AMP kinase (AMPK) and blocks malonyl-CoA production and consequently improves cellular β-oxidation. However, in the condition of an excess of plasma FFA, not only triacylglycerol (TG) accumulate inside the cells, but also other biologically active lipids: ceramide (Cer) and diacylglycerols (DAG), which were shown to interfere with the insulin signaling pathway. Because various studies have demonstrated that hepatic IRes plays a central role in the development of T2D, and obesity is centrally involved in increasing the clinical risk of diabetes, visceral adipose tissue is now thought to provide a link between obesity and hepatic IRes^10–13^.

The objective of the proposed study was to determine whether metformin treatment could reverse the insulin resistance induced by high-fat diet feeding and to gain a further understanding where external FA are directed within cells. Moreover, we sought to elucidate which bioactive lipid group plays a main role in induction of liver IRes and on which level the lipids affect insulin pathway in liver.

## Results

### HOMA-IR, OGTT and IPTT test results

**Figure 1:**
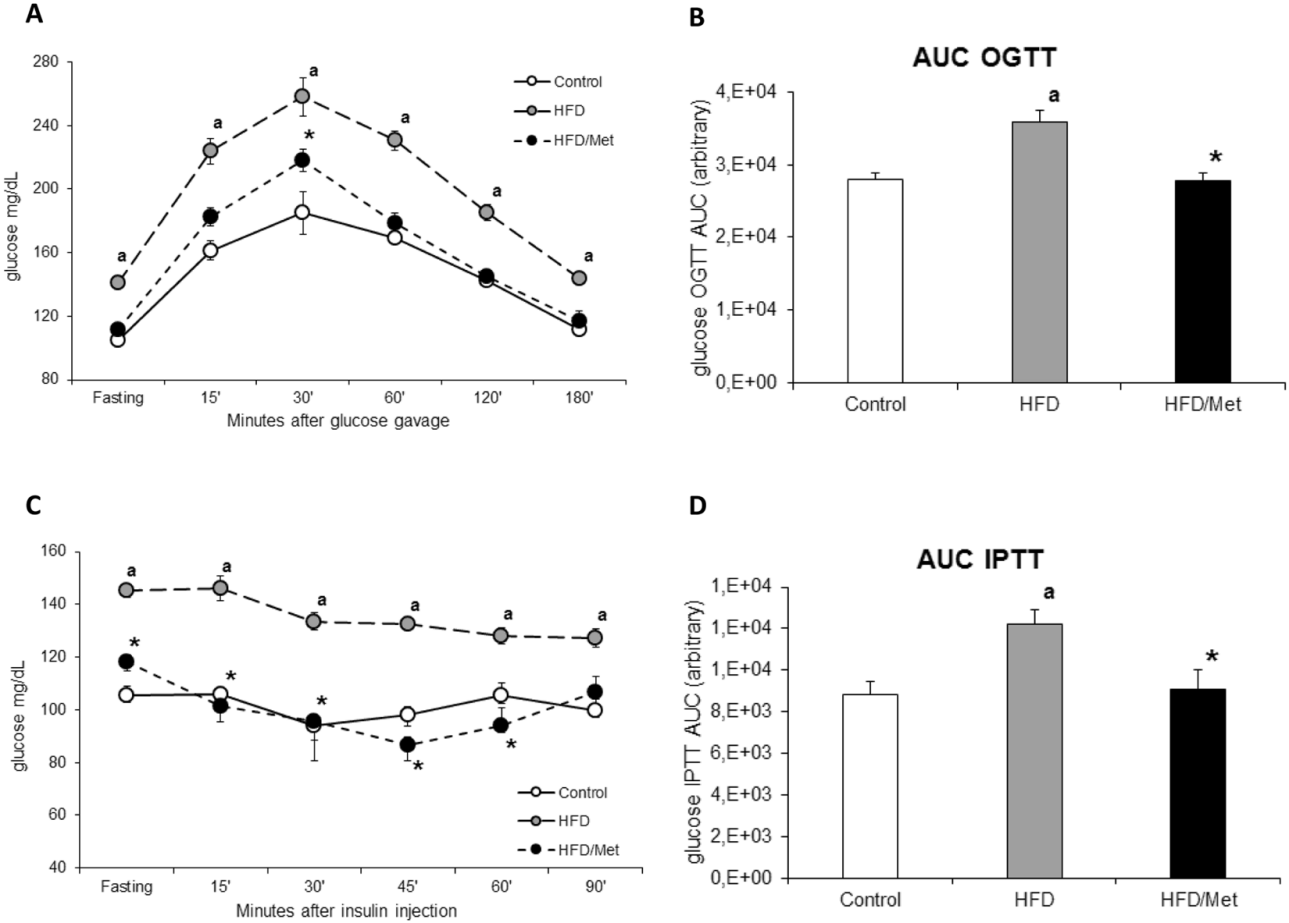
Treatment with metformin improves glucose and insulin tolerance in animals on high-fat diet. Panel A shows blood glucose profiles during oral glucose tolerance test (OGTT); Panel C shows blood glucose profiles during intraperitoneal insulin tolerance test (IPTT). Panels B and D show respective areas under blood glucose concentration curve (AUC) from OGTT and IPTT tests. Values are mean +/− SD. Symbols denote statistical significance of p < 0.05 against: a- vs. Control group; *- vs. HFD group, n = 8 per group; Significance by Anova with Tukey HSD post-hoc test.

Animals fed HFD developed IRes, as evidenced by elevated fasting blood glucose concentration, impaired glucose tolerance, reduced insulin responsiveness and increased HOMA-IR index. HOMA-IR index increased in HFD group by 46% (p < 0.05) compared to the control group (Table S1). Metformin treatment restored HOMA-IR to control value (Table S1 and Figure S2). The elevated fasting plasma glucose in HFD animals was the main factor responsible for significantly higher blood glucose profile during OGTT and IPTT test. When blood glucose value were normalized to initial fasting glucose concentration (Figure S1), the differences between control and HFD animals were significant only for IPTT. Metformin normalized insulin-related parameters and OGTT and IPTT results to the values similar to control, yet significantly different from HFD animals (Figs 1 and S1). (Table S1, Figs 1 and S1 and S2).

### Plasma FFAs and total liver TG

**Figure 2:**
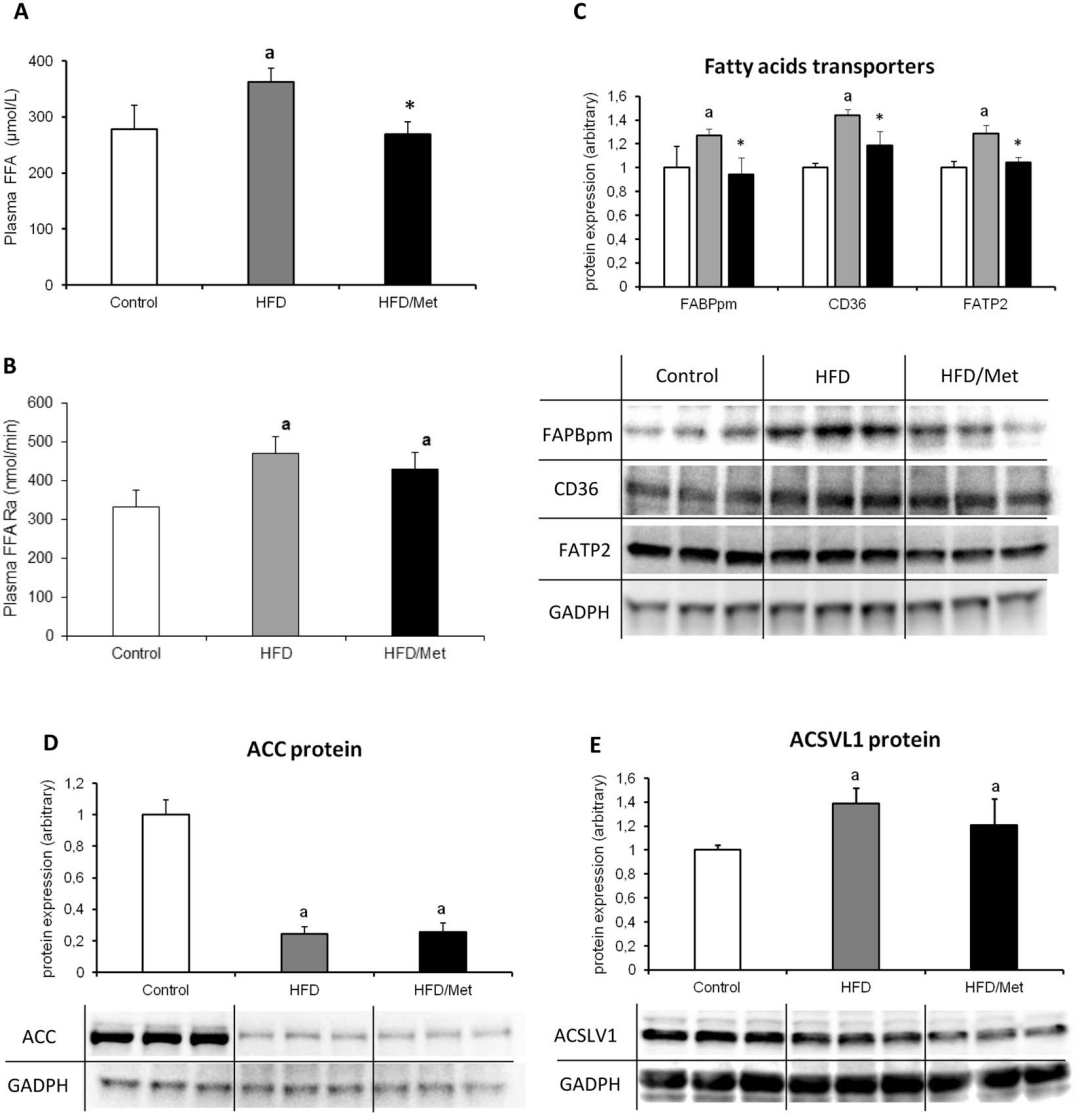
The impact of HFD consumption and metformin treatment on plasma fatty acids metabolism hepatic fatty acids transport proteins. Panel A and B present plasma fatty acid concentration and rate of appearance, respectively. Panel C presents hepatic content of plasma membrane fatty acid binding protein (FABPpm), fatty acid translocase (CD36) and fatty acid transport protein 2 (FATP2); Panel D and E present protein expression of acetyl-CoA carboxylase (ACC) and acyl-CoA synthetasease (ACSVL1) in rat liver. Values are mean +/− SD. Symbols denote statistical significance of p < 0.05 versus: a- vs. Control group; *- vs. HFD group, n = 8 per group; Significance by Anova with Tukey HSD post-hoc test.

Total plasma FFA concentration increased in HFD group by 30% (p < 0.05) as compared to the control group. The highest increase was noticed in long-chain saturated FA: C18:0, C20:0 and C24:0, whereas unsaturated FFA (C16:1 and C18:1) displayed a significant decrease (Table S2). In HFD/Met group, the total plasma FFA concentration decreased by 35% (p < 0.05) as compared to HFD group (Fig. 2A). Both HFD groups displayed increased plasma FFA rate of appearance (FFAra, Fig. 2B). Although metformin treatment managed to slightly decrease FFAra, the change was insignificant as compared to both the HFD and control. Liver TG content displayed changes analogous to plasma FFA concentration (Table S1). A high-fat diet increased liver TG level (by 67%, p < 0.05 vs Control), whereas metformin treatment decreased TG content as compared to HFD group (by 27%, p < 0.05). Despite metformin treatment, the TG content in HFD/Met group was significantly higher than the values from Control group (by 22%). (Tables S1 and S2, Fig. 2A,B).

### Fatty acid transporters

In both groups fed HFD, the content of all the measured fatty acid transporters (FABPpm, CD36, FATP2) increased as compared to control group (p < 0.05). Metformin treatment decreased protein expression of FA transporters in the liver to the values indifferent from Control. (Fig. 2C).

### Content of ACC and ACSVL1

The content of ACC decreased in both HFD groups (HFD and HFD/Met) as compared to the control group (p < 0.05). The content of ACSVL1 was significantly higher in both group fed HFD as compared to control, yet it was more visible in HFD-only animals. (Fig. 2D,E).

### Ceramide and DAG concentration and fractional synthesis rate

**Figure 3:**
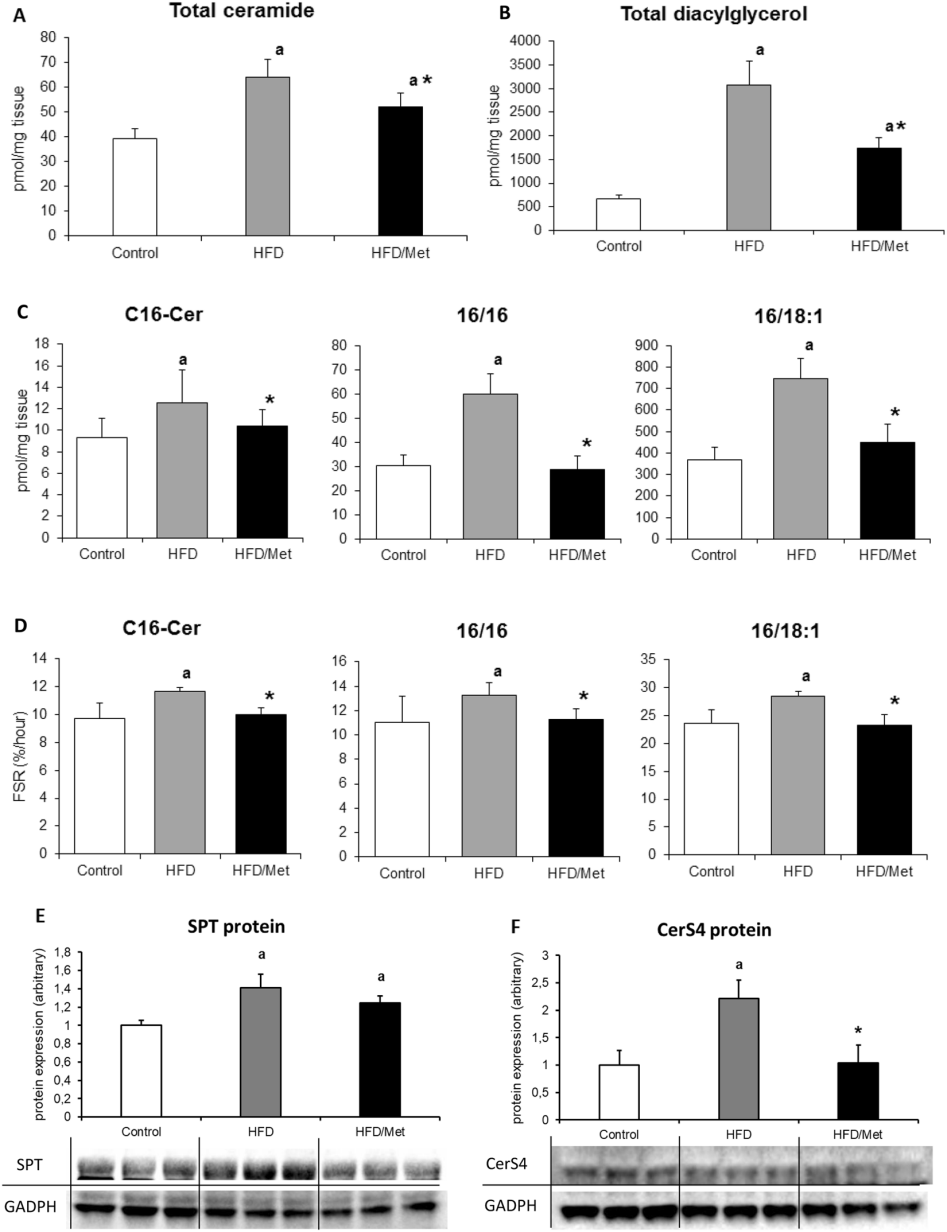
Metformin treatment normalizes hepatic content an synthesis rate of ceramide and diacylglycerol in the liver of rats fed HFD diet. Panel A and B present a total ceramide and diacylglycerol content in rat liver, respectively; Panels in the C row show the content of ceramide and diacylglycerol molecular species with established FSR. Panels in the D row present respective FSR values of the individual lipid species from the panels in the C row. Panel E and F present hepatic protein content of SPT and CerS4. Values are mean +/− SD. Symbols denote statistical significance of p < 0.05 versus: a- vs. Control group; *- vs. HFD group. n = 8 per group; Significance by Anova with Tukey HSD post-hoc test.

The total content of ceramide and DAG significantly increased in the groups fed HFD as compared to the control. Metformin treatment reduced the total content of bioactive lipids as compared to HFD group (for all p < 0,05), yet it was still significantly higher than the control value. In the HFD group the highest increase was observed in the case of C18:0-Cer and C24:0-Cer (around two times higher than in the control group, p < 0.05, Table S3). In the HFD/Met group the level of all ceramide molecular species except C18:1-Cer was significantly lower than in the HFD group (p < 0.05). The synthesis rate of C16:0-Cer significantly increased in the HFD group and returned to control values after metformin treatment. The fractional synthesis rate of ceramide reflects changes in the hepatic content of this lipid (Fig. 3C). (Fig. 3A–D, Tables S3 and S4).

In the HFD group, the content of all the measured DAG species was significantly higher as compared to the control group (p < 0.05). Metformin treatment significantly decreased the total and individual molecular species of DAG compared to HFD, yet the DAG content was still higher than the control. Exceptions were noted for C16:0/16:0-DAG and C16:0/18:1-DAG, which were normalized to control values by metformin treatment (Table S4). The synthesis rate of C16:0/16:0 and C16:0/18:1 corresponds to changes in the content of these DAG species, being significantly higher in the liver of HFD-only animals and not different than control in HFD/Met group (Fig. 3D).

### Expression of ceramide synthesis proteins

The protein content of SPT increased in both HFD groups as compared to control (p < 0.05); however, in the group treated with metformin, a slight, but insignificant, decrease of the protein level was observed. The content of CerS4 also increased in both HFD groups, but metformin treatment normalized CerS4 protein content to the control value. It could be noted that the protein expression of CerS4 corresponds to the level and synthesis rate of ceramides, which are synthesized by this enzyme isoform (e.g. ceramides acylated with 18- to 24-carbon length FA) (Fig. 3E,F).

### Acyl-carnitine and mitochondrial protein expression

**Figure 4:**
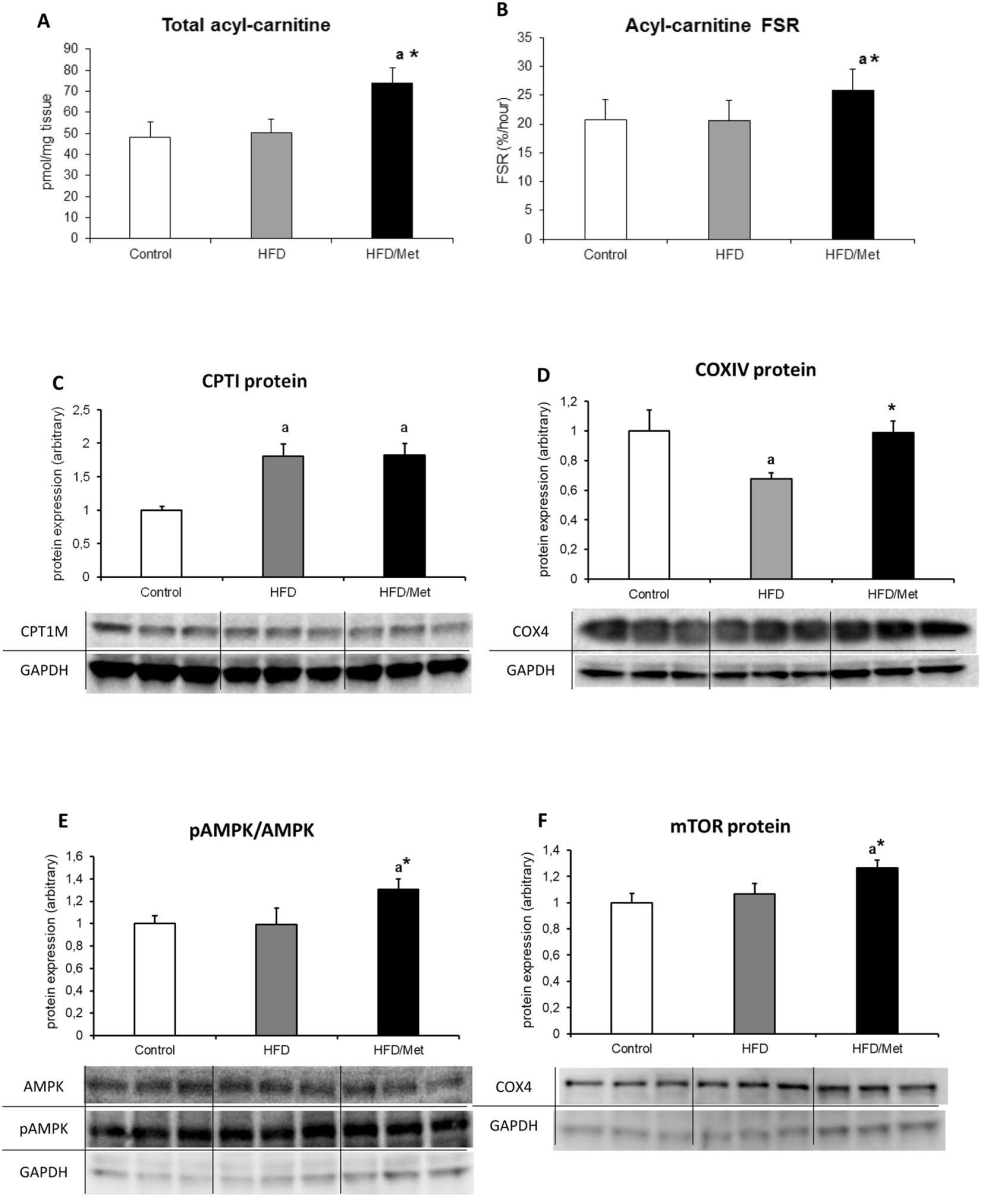
The impact of HFD consumption and metformin treatment on mitochondrial channeling of fatty acids and AMPK/mTOR proteins. Panel A and B present total content and synthesis rate of hepatic acyl-carnitine, respectively; Panel C and D show liver expression of carnitine palmitoyltransferase 1 (CPTI) and cytochrome c oxidase subunit IV (COX IV, mitochondrial marker); Panels E and F present phosphorylation state of AMPK and the protein expression of mTOR. Values are mean +/− SD. Symbols denote statistical significance of p < 0.05 versus: a- vs. Control group; *- vs. HFD group. n = 8 per group; Significance by Anova with Tukey HSD post-hoc test.

The increase in the hepatic content and synthesis rate of acyl-carnitine was noted only in HFD/Met group. Although mitochondrial CPTI FA transporter increased in liver of both HFD-treated groups (p < 0.05 vs Control), other mitochondrial protein markers and regulators displayed no significant change (mTOR and AMPK expression and phosphoryation) or decrease (COXIV, p < 0.05 vs Control) in HFD-only animals. Metformin treatment normalized COXIV expression and significantly up-regulated protein expression, phosphorylation state of AMPK and protein expression of mTOR as compared to both the Control and HFD animals. (Fig. 4A–F and Table S5).

### The impact of HFD and metformin treatment on insulin signaling cascade after *in-vivo* insulin stimulation

#### Phosphorylation state of IRS1 and AKT

**Figure 5:**
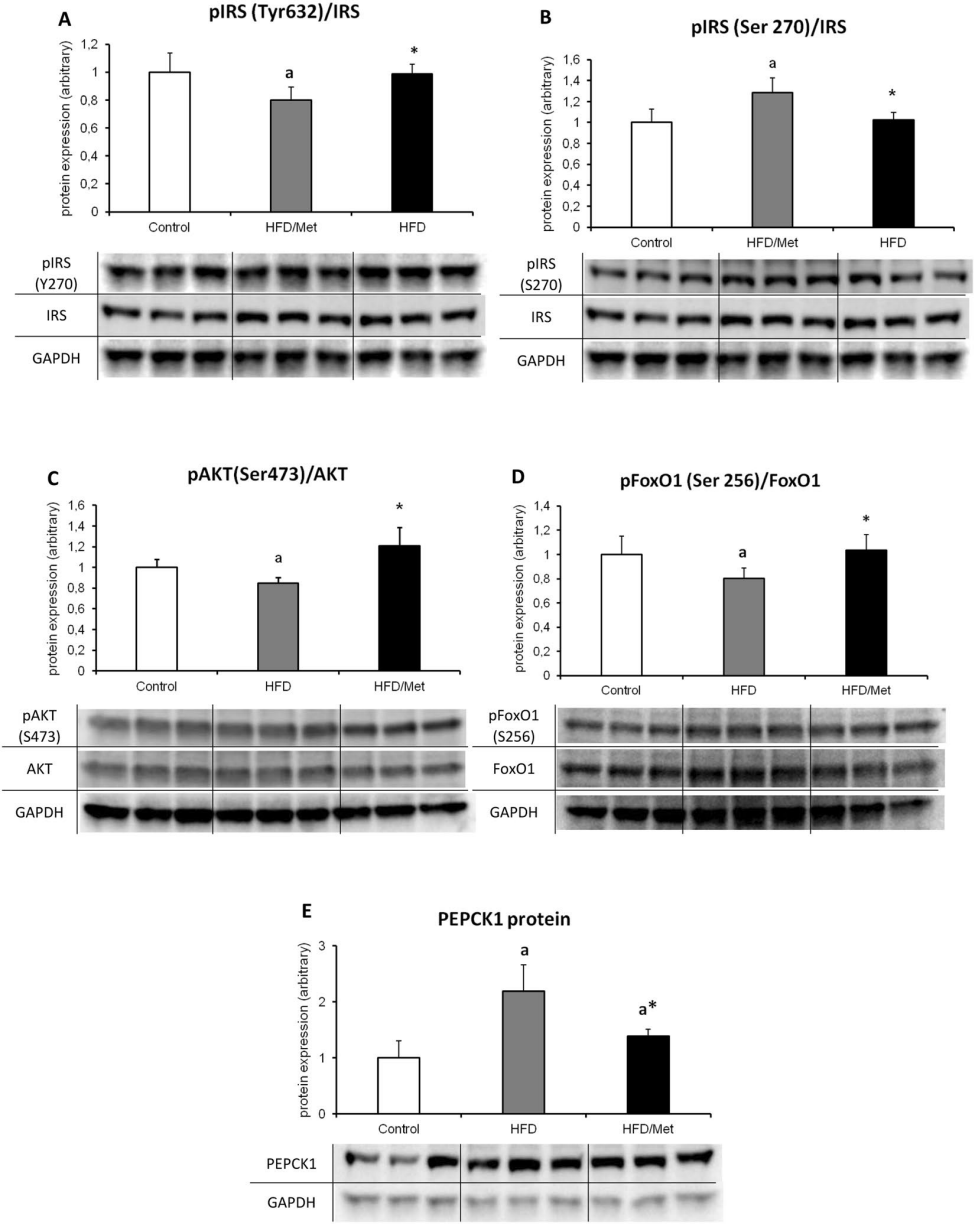
Metformin treatment of HFD animals improves hepatic insulin signaling. The figure present activatory (Tyr632, Panel A) and inhibitory (Ser270, Panel B) phosphorylation state of insulin receptor substrate 1 (IRS1); Panel C presents a ratio phosphorylation state of Akt at Ser473 and panel D shows a FoxO1 phosphorylation state at Ser256; The impact of HFD and metformin treatment on protein content of PEPCK1 is presented in Panel E. Values are mean +/− SD. Symbols denote statistical significance of p < 0.05 versus: a- vs. Control group; *- vs. HFD group. n = 8 per group; Significance by Anova with Tukey HSD post-hoc test.

The phosphorylation state of IRS1 in HFD animals as measured by the pIRS to total IRS was significantly lower for Tyr632 and significantly higher for Ser270, which suggests an inhibition of insulin cascade at the level of IRS1 protein. Metformin treatment normalized IRS phosphorylation state to the levels indifferent from control, but significantly different compared to HFD animals. The ratio of pAkt(Ser473) to unphosphorylated Akt decreased in HFD group. Metformin treatment restored the phosphorylation of Akt to control values. (Fig. 5).

#### Protein expression of FoxO1 and PEPCK1

The ratio of phosphorylated to unphosphorylated form of FoxO1 decreased in HFD group as compared to the control group, and returned to control values with metformin treatment. The results indicate that HFD treatment augmented gluconeogenesis signaling through stimulatory de-phosphorylation of FoxO1, whereas metformin inhibits hepatic gluconeogenesis through inhibitory FoxO1 phosphorylation. The hepatic PEPCK1 protein expression in HFD-only animals reflects activation of its transcriptional regulator FoxO1. A high-fat diet up-regulated PEPCK1 protein expression as compared to control. Although metformin treatment significantly decreased PEPCK1 content vs. HFD-only animals, it was unable to normalize this parameter to control values.

## Discussion

Hepatic insulin resistance is a complex metabolic disorder. Although there are many unanswered questions regarding the underlying mechanism, it is well documented, that hepatic IRes is associated with an increase in the intracellular lipid concentration^1,14,15^. Regarding the lipids, currently more emphasis is given to the impact of ceramide and diacylglycerol accumulation on insulin signaling pathway. In order to develop strategies for new therapies on IRes or T2D, it is necessary to fully understand the mechanisms by which intracellular lipid accumulation affects hepatic insulin sensitivity. In our work we have demonstrated that IRes induced by HFD feeding was manifested by a significant set of metabolic alternations such as elevated blood glucose, plasma insulin, HOMA-IR and glucose and insulin intolerance. This state was associated with increased plasma FFA concentration and turnover, especially in the case of the long-chain fatty acids (Table S2). In the group fed HFD and treated with metformin, we have not observed any signs of IRes or elevated plasma FFA concentration despite increased plasma FFA turnover as measured by tracer infusion technique. As mentioned in the introduction, FA enter the cell through fatty acid transporters. In our study, we have observed, that in the HFD group, the content of fatty acid transporters was elevated, which suggests that in these animals more FA are directed into a cell. Another source of intracellular FA is *de novo* synthesis. One of the most critical step in FA *de novo* synthesis is a reaction catalyzed by ACC. It is well documented, that metformin inhibits ACC by activation of AMPK and in this way inhibits malonyl-CoA production that is a CPT1 inhibitor and thereby is also an inhibitor of β-oxidation. In our study, the content of ACC decreased in both HFD groups yet only the HFD/Met animals displayed increased phosphorylation of AMPK. This suggest that *de-novo* synthesis of FFA is strongly inhibited by metformin. Inside a cell, FA are activated to acyl-CoA by acyl-CoA synthetase (ACSVL1). We found, that in both HFD groups content of ACSVL1 was elevated. Acyl-CoA might be used as an energy source in β-oxidation or can be used as a substrate for synthesizing of complex lipids. In the present study, we have demonstrated that HFD leads to Cer and DAG accumulation whereas metformin treatment of animals fed HFD significantly reduced the content of both ceramide and DAG but increased acyl-carnitine level. We have also noted, that content of SPT and CerS4 (the enzymes implicated in *de novo* ceramide synthesis) increased in both HFD group, but in the group treated with metformin, the amount of CerS4 was much lower than in HFD group. This enzyme is responsible for attachment of a long-chain FA (C18–C24) to sphingoid base and production of particular ceramide species in *de novo* synthesis pathway. In our study we found, that the pattern of individual ceramide species concentration relates to the CerS4 content. Despite years of intensive investigation, there was still an unanswered question, what was the origin of the accumulated hepatic lipids. In our work, with the use of stable isotope-labeled fatty acid infusion, we were able to measure not only the level of these lipids but also isotopic enrichment for the calculation of fractional synthesis rate. In our study we have demonstrated, for the first time, that in insulin resistant liver, fractional synthesis rate of both the ceramide and DAG is related to plasma fatty acids supply. Cer and DAG FSR significantly increased in the HFD group and was normalized with metformin treatment. Our data indicate that intracellular lipid-lowering effects of metformin are related to augmentation of mitochondrial channeling of fatty acids. Metformin treatment enhances mitochondrial β-oxidation process, therefore the excess of intracellular FA are directed towards β-oxidation, which decreases substrate supply for the synthesis of bioactive lipids that would affect the insulin signaling pathway. In our work we have observed that metformin treatment increased phosphorylation state of AMPK, mTOR protein expression and mitochondrial marker expression (COXIV) as compared to the HFD-only animals. Augmentation of mitochondrial biogenesis signaling by metforminę is consisted with observed elevation of CPT1 protein expression, acyl-carnitine content and acyl-carnitine synthesis rate in the HFD/Met group.

Previously published data^16–18^ and our own results indicate that cellular lipids accumulation is related with the inhibition of insulin signaling pathway. The most critical steps in activation of the insulin signaling cascade are tyrosine phosphorylation of IRS and in turn activation of phosphatidylinositol-4,5-bisphosphate 3-kinase kinase (PI3K) that results in activation of Akt^19,20^. Akt in turn catalyses FoxO1 phosphorylation that leads to nuclear exclusion of FoxO1 and in down-regulation of gluconeogenesis-related genes^21^. It has been demonstrated that phosphorylation of IRS on Ser/Thr residue causes inhibitory signal on insulin-signaling pathway, whereas the phosphorylation on Tyr residue causes activation of this cascade. In our work, we have found that HFD inhibits insulin pathway which was reflected by increased pIRS-1/2(Ser270) phosphorylation, decreased pIRS1(Tyr632) phosphorylation, decreased pAkt and pFoxO1 phosphorylation and up-regulation of PEPCK1 protein, while metformin exerted the opposite effect. The inhibitory effect of DAG on liver insulin cascade was reported by various groups^1,16,18^. It has been shown that both saturated and unsaturated fats lead to hepatic diacylglycerols accumulation, activation of PKCε, and impairment of insulin-stimulated IRS signaling at the level of IRS^22–24^. Knocking down expression of PKCε protects the animals from lipid-induced hepatic IRes^16^. Moreover it has been found that activation other isoform of PKC – PKCθ – is implicated in inhibition of insulin-signaling pathway in the liver of lipid-infused rats, which associated with three-fold increase in intracellular DAG concentration^25^. In line with those findings, our results indicate that HFD consumption lead to accumulation of intracellular DAG molecular species, which was accompanied with the pIRS-1/2(Ser270) phosphorylation and pIRS1(Tyr 632) de-phosphorylation. Metformin treatment normalized both the hepatic level of DAG and restored the IRS phosphorylation state to control values. Activation of Akt by IRS plays a central role in mediating many insulin actions by regulating the expression and activity of a wide range of enzymes and transcription factors^26^. The growing body of evidence points to the ceramide-mediated inhibition of Akt phosphorylation through the activation of protein phosphatase 2A^27,28^. In the literature, there are conflicting data regarding the role of ceramide in induction of hepatic IRes. Galbo *et al*., did not observed hepatic ceramide accumulation in animals fed HFD which suggest, that ceramide accumulation is not a major player in the development of lipid-induced hepatic IRes^22^. However, a recent lipidomic study of obese humans demonstrated strong relationship between hepatic ceramides and HOMA-IR index^29^. In our study, we showed that HFD consumption is connected with hepatic ceramide accumulation and a decrease in the phosphorylation state of Akt at Ser473 in HFD animals. Because Akt phosphorylation at Ser473 is required for activation of Akt kinase, our results indicate, that HFD causes inhibition of insulin signaling also at the level of Akt, presumably due to hepatic ceramide accumulation. Metformin treatment decreases ceramide content and restores the phosphorylation state of Akt to control value. As mentioned earlier, FoxO family of transcription factors controls the expression of lipogenic and gluconeogenic genes. As in the case of AMPK, FoxO1 is directly phosphorylated by Akt, which leads to the exclusion of FoxO1 from the nucleus and blocking of its transcriptional activity. In conditions of impaired insulin signaling through both the AMPK and Akt inhibition, FoxO1 activity increases, leading to excessive glucose production. Our results show, that HFD consumption is connected with both the Akt inhibition and decrease in the phosphorylation state of FoxO1 protein.

Taken together, HFD induced IRes, triggered hepatic accumulation of both the ceramide and DAG content and inhibited insulin signaling, whereas metformin treatment improved insulin sensitivity, decreased the content of ceramide and DAG and augmented hepatic insulin signaling. Beneficial changes of metformin treatment were connected with increased mitochondrial lipid channeling as indicated by increased content of acyl-carnitine and mitochondrial markers what was accompanied with insulin cascade activation. We hypothesize that the induction of IRes is a cumulative effect of the active lipids accumulation. Moreover, in the liver, the insulin sensitizing effect of metformin consists in an enhanced β-oxidation process that protects hepatic cells from active lipid accumulation that would affect insulin pathway. Additionally, we have answered for a question regarding the origin of accumulated lipids, and have demonstrated that the changes in lipids corresponds to the *de novo* synthesis.

## Material and Methods

### Animals and study design

The investigation was approved by the Institutional Animal Care and Use Committee of the Medical University of Bialystok. All methods were performed in accordance with the relevant guidelines and regulations. The experiments were carried out on male Wistar rats (140–150 g), randomly divided into the following groups (n = 8 in each group): 1. C - control group fed ad libitum a control diet (Research Diets INC D12450B). 2. HFD - group fed high-fat diet (Research Diets INC D12492). 3. HFD/MET - group fed high-fat diet with oral dose of metformin (300 mg/kg). The control diet contained 10% kcal from fat, The HFD contained 60% kcal from fat. All groups were fed for eight weeks with appropriate chow. Every week the plasma insulin and glucose concentration was checked for HOMA-IR calculation, and the HFD/MET group started metformin treatment at the fifth week of HFD consumption after the induction of whole body insulin resistance (Figure S2). During the last week of the experiment an oral glucose tolerance test (OGTT) and an intraperitoneal insulin tolerance test (IPTT) were undertaken on fasted animals (six hour fast). On the last day of the study, the food was withdrawn six hours before the start of the infusion protocol. The [U-^13^C]palmitate was infused into a proximal dorsal tail vein^30^, using a syringe pump (New Era Syringe Pumps, Farmingdale, NY, USA) via 0.5 mm I.D. tubing and 25 gauge needle at a constant rate of 50 nmol/min^−1^/kg^−1^ body weight for two hours. An albumin-bound [U-^13^C]palmitate tracer was prepared as previously described^31^. A priming dose of ^13^C_16_-potassium-palmitate (500 nmol/kg) was given in the first ten seconds of infusion to prime palmitate pool and accelerate isotopic equilibration. Every 15 minutes a 50 μL blood sample was taken into heparinized Microvette capillary tube (Stardstedt, Numbrecht, Germany). The mean plasma [U-^13^C]palmitate enrichment was identical in all experimental groups (Table S1), which was the result of adjusting the infusion rate for the weight of the animal. To prevent vascular volume overload, the infused volume did not exceeded 17% of total plasma volume (calculated according to Lee *et al*.^32^) and was less than estimated 2-hour urine output according to the values for Wistar rats from Rat Phenome Database^33^. Half an hour before finishing the infusion, insulin (0.5 U/kg) was administrated intraperitoneally to measure the insulin-stimulated protein phosphorylation in insulin cascade. The rats were anaesthetized by intraperitoneal injection of pentobarbital in a dose of 80 mg/kg of body weight. The liver was taken and frozen in liquid nitrogen and then stored at −80 °C until analysis.

### Measurements of lipid conentration

Plasma FFA concentration and isotopic enrichment were measured by LC/MS according to Persson *et al*.^34^. Fatty acids were separated on the LC using a reverse-phase Zorbax SB-C18 column 2.1 × 150 mm, 1.8 µm, using two buffers. Buffer A was 80% acetonitrile, 0.5 mM ammonium acetate; buffer B was 99% acetonitrile, 1% 0.5 mM ammonium acetate. The ceramide content and isotopic enrichment was measured with the use of an UHPLC/MS/MS approach according to Blachnio-Zabielska *et al*.^35^. Sphingolipids content and isotopic ceramide enrichment were analyzed by means of a triple quadrupole mass spectrometer using positive ion electrospray ionization (ESI) with multiple reaction monitoring (MRM) against the concentration and enrichment standard curves. The content and isotopic enrichment of DAG were measured using a UHPLC/MS/MS approach according to Blachnio-Zabielska *et al*.^36^. The following DAG were quantified: C18:1/18:2, C16:0/18:2, C16:0/16:0, C16:0/18:1, C18:0/20:0, C18:0/18:1, C18:1/18:1, C18:0/18:2 and C16:0/18:0 using UHPLC/MS/MS. Isotopic enrichment was analyzed in the C16/16 and C16/18:1. Diacylglycerols content and isotopic enrichment were analyzed against the concentration and enrichment standard curves. Acyl-carnitine concentration and isotopic enrichment (^13^C16-carnitine) were measured according to Sun *et al*.^37^. Liver triacylglycerol (TG) content was measured with the use of Triglyceride Quantitation Kit (Sigma Aldrich, St. Louis, MO) and Varioscan Lux Multimode Microplate Reader (ThermoFisher Scientific, Waltham, MA), according to manufacturer guidelines.

### Western Blot

The following target proteins were quantified using primary antibodies: GOT2 (FABPpm): (Novus Biologicals), CPTI; CD36; ACSVL1; FATP2; LASS4 (CerS4); IRS-1; p-IRS-1/2 (Ser 270): p-IRS-1 (Tyr632), GAPDH (Santa Cruz Biotechnology): SPT; AMPK; pAMPK (T183 and T172) (Abcam), FoxO1; pFoxO1(Ser256); COX IV, mTORPEPCK1 (Cell Signaling) and appropriate HRP conjugated secondary antibodies. The proteins were blotted onto PVDF membranes using semi-dry transfer and the expression was measured by chemiluminescence using ChemiDoc XRS+ system (Hercules, CA) and ImageLab software. Values were normalized to GAPDH protein expression measured from the same run and expressed as fold changes over control group values. Unless stated otherwise, all chemicals and equipment used for immunoblotting were purchased from Bio-Rad (Hercules, CA).

### Oral Glucose Tolerance Test (OGTT)

OGTT in fasted (6 h) animals was performed as follows: blood glucose was measured before and after 15, 30, 60, 120 and 180 minutes after orally glucose administration in a dose of 3 g/kg. Blood samples from the tail veins were measured by using glucometer Accuchek (Roche. Germany). The area under the plasma glucose curve for OGTT was calculated using trapezoidal rule for both the original (Fig. 1) and fasting glucose normalized values (Figure S2).

### Intraperitoneal Insulin Tolerance Test (IPTT)

Fasted (6 h) animals received an intraperitoneal injection of insulin in a dose of 0.75U/kg body weight. Glucose concentration was measured on samples obtained from the tail vein using glucometer Accuchek (Roche. Germany) at 0, 15, 30, 45, 60 and 90 minutes after insulin injection. The area under the plasma glucose curve for IPTT was calculated using trapezoidal rule for both the original (Fig. 1) and fasting glucose normalized values (Figure S2).

### Plasma glucose and insulin concentration

Plasma glucose was determined using Accuchek glucometer (Roche. Germany). Plasma insulin was measured with an ELISA insulin assay (Rat/Mouse Insulin kit, Millipore).

### HOMA-IR

HOMA-IR index value was calculated according to formula^38^:

$$\text{HOMA} \mbox{-} \text{IR}=[{\rm{fasting}}\,{\rm{glucose}}\,({\rm{mg}}/{\rm{dl}})\times {\rm{fasting}}\,{\rm{insulin}}\,({\rm{lU}}/{\rm{ml}})]/2430$$

#### Protein concentration

The protein content in homogenates was measuredmeasured with the reducing agent compatibile BCA protein assay kit. Bovine serum albumin (fatty acid free) was used as a protein concentration standard.

### Statistical analysis

Statistical significance between groups was estimated using ANOVA with the Tukey honestly significant difference (HSD) post-hoc test for equal n-numbers (n = 8). Significance level was set to p < 0.05.

## Electronic supplementary material

Supplementary Information
