# Supplementary material for: The effect of high fat diet and metformin treatment on liver lipids accumulation and their impact on insulin action
Source: Sci Rep. 2018 May 8;8:7249. doi: 10.1038/s41598-018-25397-6 (PMC5940807; doi:10.1038/s41598-018-25397-6)

The effect of high fat diet and metformin treatment on the individual lipids accumulation and their impact on insulin action in liver.

Piotr Zabielski[1,2], Hady Razak Hady[3], Marta Chacinska[2,4], Kamila Roszyc[4], Jan Górski[2], Agnieszka U. Blachnio-Zabielska[2,4]*

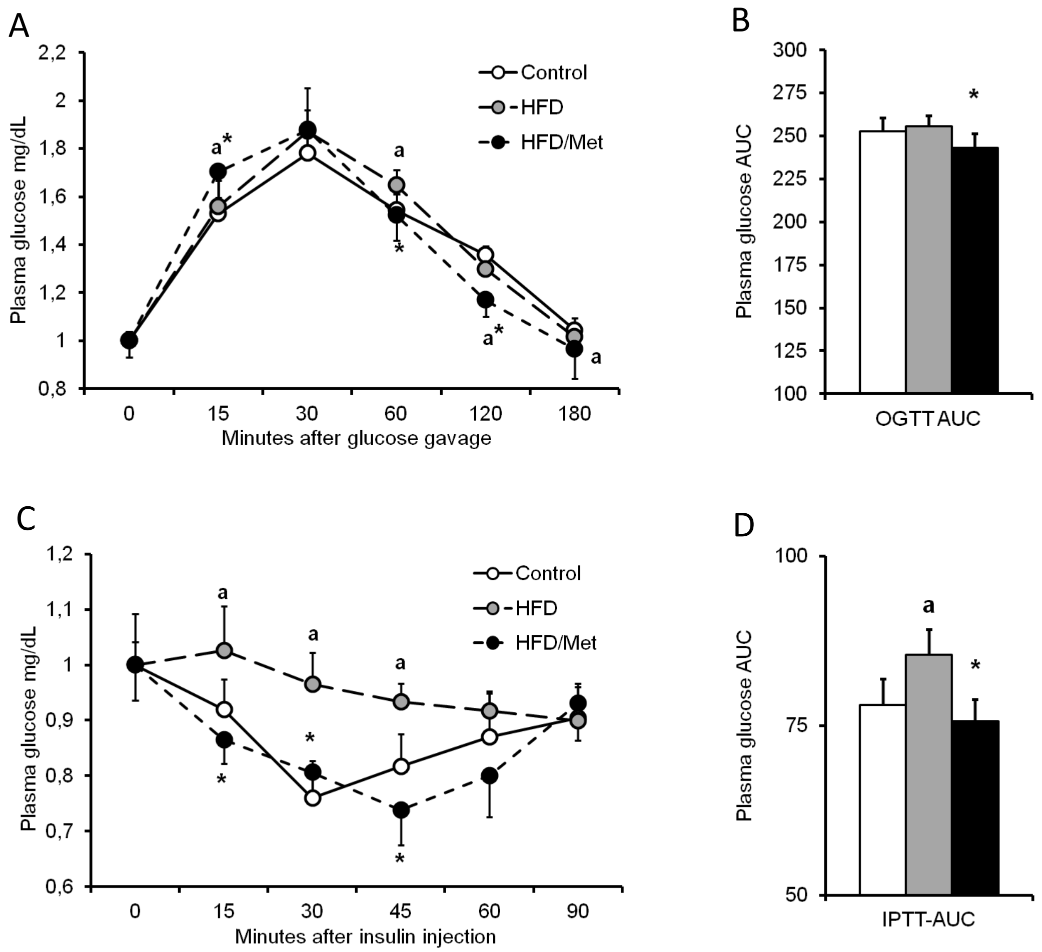

Figure S1. Normalized blood glucose profiles and their respective area under the curve (AUC) during oral glucose tolerance test (OGTT, panels A and B) and intraperitoneal insulin tolerance test (IPTT, panels C and D). The values were normalized to the initial fasting plasma glucose concentration to level the differences introduced by significantly higher fasting glucose in HFD animals. Values are mean +/- SD, n=8 per group. Significance by ANOVA (post-hoc Tukey HSD test), a – p<0.05 vs Control; * - p<0.05 vs HFD group.

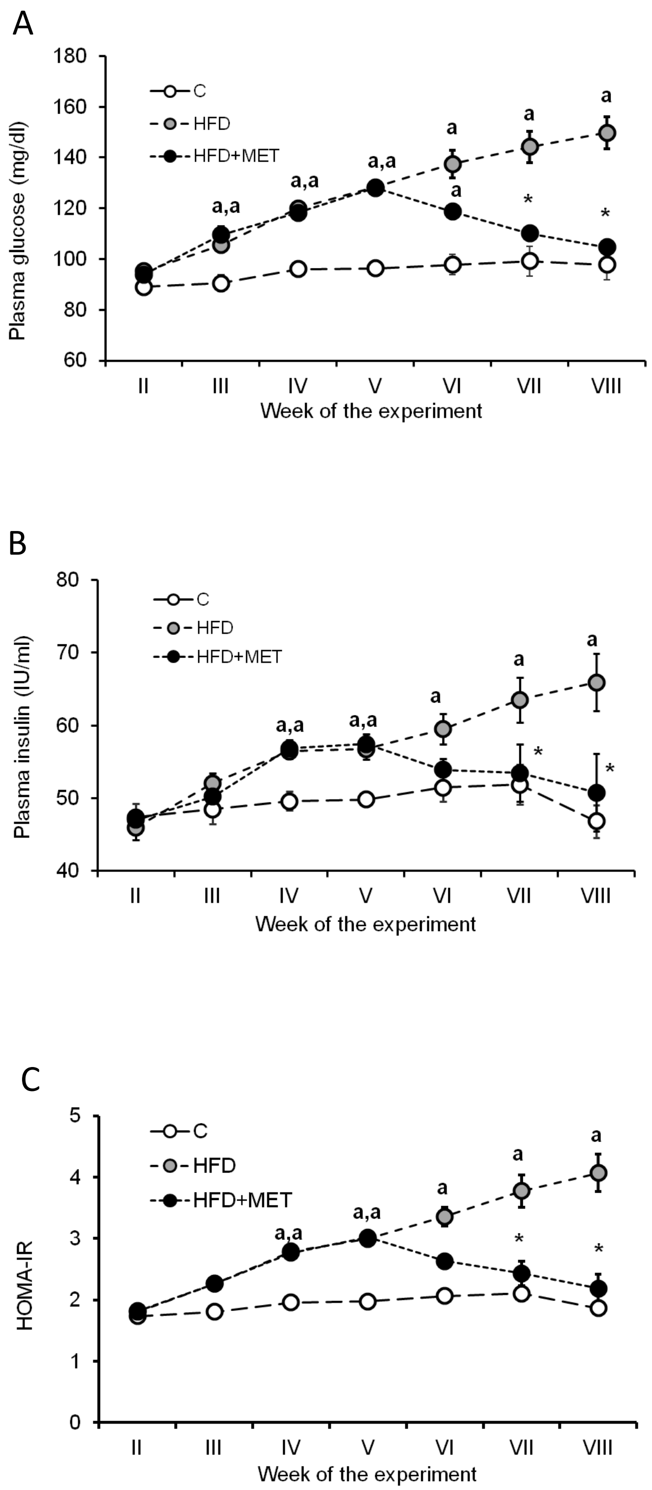

Figure S2. The impact of high-fat diet (HFD) and subsequent metformin treatment on plasma glucose concentration (Panel A), plasma insulin (Panel B) and HOMA-IR (Panel C) over the course of 8 weeks of HFD consumption. The metformin was introduced to HFD animals at 5th week of the experiment. Values are mean +/- SD, n=8 per group. Significance by ANOVA (post-hoc Tukey HSD test), a – p<0.05 vs Control; * - p<0.05 vs HFD group.

Table S1. Anthropometric parameters.

	Control	HFD	HFD/Met
Fasting plasma glucose concentration [mg/dL]	97.8±14.3	139.2±27.1[a]	104.6±4.6[*]
Fasting insulin concentration [μU/ml]	51.7±12.9	65.9±9.6	50.8±15.1
HOMA-IR	2.08±0.61	3.75±0.84[a]	2.19±0.67[*]
Plasma FFA concentration [μmol/L]	278.1±42.4	362.9±23.5[a]	268.7±21.9[*]
Plasma [U-^{13}C] palmitate enrichment [MPE]	0.252±0.025	0.254±0.007	0.259±0.010
Initial body weight (g)	149±4.7	149.5±4.6	150.7±4.7
Final body weight (g)	357.2±35.5	399.6±17.0[a]	355.2±26.7[*]
Liver triacylglycerols (nmol/mg)	12.49±1.25	20.94±2.55[a]	15.23±1.60[a*]

a – p<0.05 vs Control; *- p<0.05 vs HFD; Significance by Anova with Tukey post-hoc test; n=8 per group

Table S2. Plasma FFA concentration.

	C14:0	C16:1	C18:2	C16:0	C18:1	C18:0	C20:0	C22:0	C24:1	C24:0
Control	12.9±2.8	7.5±1.8	66.2±14.3	59.9±10.1	64.9±15.5	35.4±5.1	1.0±0.2	4.7±0.8	1.8±0.4	23.7±3.3
HFD	15.5±2.8	2.1±0.5[a]	59.9±3.2	60.7±4.9	45.6±8.7[a]	91.4±10.5[a]	4.3±0.9[a]	11.7±0.9[a]	2.3±0.5[a]	69.4±15.6[a]
HFD/Met	15.7±3.1	2.0±0.5[a]	54.6±11.4	44.1±5.2[a]*	29.4±4.2[a]*	56.1±9.9[a]*	2.8±0.7[a]*	11.4±2.0[a]	4.4±0.7[a]*	48.0±8.5[a]*

a – p<0.05 vs Control; *- p<0.05 vs HFD; Significance by Anova with Tukey post-hoc test; n=8 per group

Table S3. The liver content of individual molecular species of ceramide (Cer).

	C14:0-Cer	C16:0-Cer	C18:1-Cer	C18:0-Cer	C20:0-Cer	C22:0-Cer	C24:1-Cer	C24:0-Cer
Control	0.08±0.009	9.3±1.8	0.03±0.009	0.7±0.1	0.5±0.08	4.1±0.5	6.0±0.8	18.6±3.0
HFD	0.04±0.006[a]	12.6±3.0[a]	0.04±0.01	1.4±0.13[a]	0.8±0.08[a]	5.9±0.6[a]	6.2±0.9	36.8±5.5[a]
HFD/Met	0.03±0.004[a]*	10.4±1.5*	0.04±0.005	1.1±0.25[a]*	0.65±0.07[a]*	5.0±0.5[a]*	4.8±0.7[a]*	30.0±5.2[a]*

a – $p<0.05$ vs Control; *- $p<0.05$ vs HFD; Significance by Anova with Tukey post-hoc test; n=8 per group

Table S4. The liver content of individual molecular species of diacylglycerol (DAG).

	C16:0/C16:0 DAG	C16:0/C18:1 DAG	C16:0/C18:2 DAG	C16:0/C18:0 DAG	C18:1/C18:1 DAG	C18:1/C18:2 DAG	C18:0/C18:1 DAG
Control	30.4±4.4	367.4±59.4	142.1±26.4	20.1±4.8	62.0±12.6	53.5±11.0	1.04±0.17
HFD	60.0±8.4[a]	745.4±95.4[a]	684.4±120.3[a]	191.9±36.0[a]	682.2±102.1[a]	691.7±119.4[a]	7.6±1.13[a]
HFD/Met	28.7±5.6*	448.0±84.5*	380.0±48.4[a]*	61.0±10.6[a]*	457.6±72.8[a]*	371.6±50.6[a]*	1.8±0.45[a]*

a – $p<0.05$ vs Control; *- $p<0.05$ vs HFD; Significance by Anova with Tukey post-hoc test; n=8 per group

Table S5. Hepatic content of acyl-carnitine.

	Control	HFD	HFD/Met
**C16-Carnitine**	36.2 ± 5.2	37.4±4.6	53.5±5.8*
**C18:1-Carnitine**	11.8±2.9	12.8±2.6	20.3±3.3*

a – p<0.05 vs Control; *- p<0.05 vs HFD; Significance by Anova with Tukey post-hoc test; n=8 per group

Supplementary Materials and Methods:

Calculation of fractional synthesis rate:

Fractional synthesis rate of palmitoyl-carnitine. diacylglycerol and palmitoyl- ceramide was calculated by the tracer incorporation method. which is based on the precursor-product principle. using the following equation:

$$FSR(\%/h) = [eB(t_{120}) - eB(t_0)]\times 60\times 100\% / t0\int t_{120} eA\Delta t$$

Where:

$eB(t_{120})$ – enrichment at t120min. $eB(t_0)$ – enrichment before labeled palmitate infusion. 60 – hour in minutes. FSR – fractional synthesis rate %/hour. eA – plasma palmitate enrichment (precursor pool)

Δt – delta time from t0 to t120 (in minutes). $t0\int tx\ eA\Delta t$ - area under plasma palmitate precursor enrichment curve. integral of enrichment versus time function. Values t120; eB(t120) and $t0\int tx\ eA\Delta t$ were calculated individually for each animal and analyzed tissue. Basal enrichment ($eB(t_0)$) in all the measured lipid classes was established prior to infusion in 3 isotope-naïve animals from each of the experimental group.

Measurement of lipid concentration.

Sphingolipids

The ceramide content and isotopic enrichment was measured using a UHPLC/MS/MS approach according to Blachnio-Zabielska et al. [1]. Briefly. the liver samples (~20 mg) was pulverized and after that homogenized in a solution composed of 0.25 M sucrose. 25 mM KCl. 50 mM Tris and 0.5 mM EDTA. pH 7.4. Immediately afterwards. the internal standard (17C-sphingosine. 17C-S1P. d17:1/8:0. d17:1/18:0. d17:1/18:1(9Z). d17:1/20:0. d17:1/24:0 and d17:1/24:1(15Z) (Avanti Polar Lipids. Alabaster. Al) as well as extraction mixture (isopropanol:water:ethyl acetate. 30:10:60; v:v:v) was added to each sample. The mixture was vortexed. sonicated and then centrifuged. The supernatant was transferred to a new tube and pellet was re-extracted. After centrifugation supernatants were combined and evaporated under nitrogen. The dried sample was reconstituted in LC Solvent A (2 mM ammonium formate. 0.15 % formic acid in methanol) for UHPLC/MS/MS analysis. Sphingolipids content and isotopic ceramide enrichment were

analyzed by means of an triple quadrupole mass spectrometer using positive ion electrospray ionization (ESI) source with multiple reaction monitoring (MRM) against the concentration and enrichment standard curves respectively.

Diacylglycerols

The content and isotopic enrichment of DAG was measured using a UHPLC/MS/MS approach according to Blachnio-Zabielska et al [2]. Diacylglycerols was extracted together with sphingolipids. A known amount of internal standard mix (Deuterated DAG Mixture I and Mixture II – Avanti Polar Lipids) was added to each sample. Next. samples were extracted as described above. The following DAG were quantified: C18:1/18:2. C16:0/18:2. C16:0/16:0. C16:0/18:1. C18:0/20:0. C18:0/18:1. C18:1/18:1. C18:0/ 18:2 and C16:0/18:0 using UHPLC/MS/MS. Isotopic enrichment was analyzed in the 16/16 and 16/18:1. Diacylglycerols content and isotopic enrichment were analyzed by means of a triple quadrupole mass spectrometer using positive ion electrospray ionization (ESI) source with multiple reaction monitoring (MRM) against the concentration and enrichment standard curves respectively.

Acyl-carnitines

Acyl-carnitine concentration and isotopic enrichment (^{13}C16-carnitine) was measured according to Sun et al. [3] with minor modification. Both. concentration and isotopic enrichment were measured with the use of UHPLC/MS. Briefly. frozen liver sample (20–30 mg) were pulverized into a fine powder under liquid nitrogen. 50µl of freshly made 1 M KH_2PO_4 and a C17-carnitine as an internal standard was added prior to extraction procedure to each sample. Next. samples were extracted with freshly made extraction solution of 3:1 ACN/MeOH (v/v) and centrifuged at 14.000 ***g*** for 20 min at 4°C. The supernatant was transferred to fresh tube and dried under N_2. Next. 100µl of 3:1 ACN/MeOH was added and vortex for 5 min and sonicated for 15 min. The sample was then centrifuged at 14.000 ***g*** for 20 min at 4°C. and the clear solution was carefully transferred into analytical vials for LC/MS analysis.

Plasma FFA

Plasma FFA concentration and isotopic enrichment was measured by LC/MS according to Persson et al [4]. Briefly. the concentrations of FFA were measured against a six point standard

curve and the isotopic enrichment (^{13}C16) against an eight-point (0.00%–0.40%) enrichment curve. The standards and plasma samples were extracted with freshly prepared Dole solution composed of isopropanol: heptanes:1 M H_2SO_4 (40:10:1; v/v/v). Prior the extraction procedure a known amount of heptadecanoate was added as an internal standard. The extracts were allowed to dry under nitrogen. The dried samples were resuspended in a buffer A for the the LC/MS. Fatty acids were separated on the LC using a reverse-phase Zorbax SB-C18 column 2.1 x 150 mm. 1.8 μm. using two buffers. Buffer A is 80 % acetonitrile. 0.5 mM ammonium acetate; buffer B was 99 % acetonitrile. 1 % 0.5 mM ammonium acetate.

Liver triacylglycerols:

The total content of liver triacylglycerols was measured with the use of Trigliceride Quantitation Kit (Sigma Aldrich. St. Louis. MO) according to manufacturer guidelines. Briefly. approx. 25mg of liver tissue was homogenized for 30 seconds in ice-cold 5% Trion X-100 (1:10. w/v) with the use of Vibra Cell VCX 130 PB sonicator (Sonics&Materials. Newtown. CT). The homogenate was incubated for 2 minutes in 100°C to solubilize cellular lipids. After centrifugation supernatant was diluted 10 times. TG content was quantified in using sample duplicates. after liberation of TG-bound glycerol by lipase mix incubation with subsequent labeling with fluorescent probe. Sample fluorescence (λex = 535/λem =590 nm) was measured with the use of Varioscan Lux Multimode Microplate Reader (ThermoFisher Scientific. Waltham. MA) against freshly-prepared TG standard curve.

1 Blachnio-Zabielska. A. U.. Persson. X. M.. Koutsari. C.. Zabielski. P. & Jensen. M. D. A liquid chromatography/tandem mass spectrometry method for measuring the in vivo incorporation of plasma free fatty acids into intramyocellular ceramides in humans. *Rapid Commun Mass Spectrom* **26**. 1134-1140. doi:10.1002/rcm.6216 (2012).

2 Blachnio-Zabielska. A. U.. Zabielski. P. & Jensen. M. D. Intramyocellular diacylglycerol concentrations and [U-13C]palmitate isotopic enrichment measured by LC/MS/MS. *J Lipid Res* **54**. 1705-1711. doi:10.1194/jlr.D035006 (2013).

3 Sun. D.. Cree. M. G.. Zhang. X. J.. Børsheim. E. & Wolfe. R. R. Measurement of stable isotopic enrichment and concentration of long-chain fatty acyl-carnitines in tissue by HPLC-MS. *J Lipid Res* **47**. 431-439. doi:D500026-JLR200 [pii] 10.1194/jlr.D500026-JLR200 (2006).

4 Persson. X. M.. Blachnio-Zabielska. A. U. & Jensen. M. D. Rapid measurement of plasma free fatty acid concentration and isotopic enrichment using LC/MS. J *Lipid Res* 5**1.** 2761-2765. doi:jlr.M008011 [pii] 10.1194/jlr.M008011 (2010).

All western blots were performed as described below.
"Exposure Time (sec)" was individually set for particular proteins.

The sequence of application the samples into the gel wells:
1st lane – Protein standard
2nd lane – Control sample
3rd lane – HFD sample
4th lane – HFD/Met sample
5th and 6th land – other samples (not analyzed)
7th lane – Control sample again
8th lane – HFD sample again
9th lane – HFD/Met sample again
10th and 11th land – other samples (not analyzed)
12th lane… repeats according to the system described above

Acquisition Information

Imager	ChemiDoc XRS+
Exposure Time (sec)	6.784 (Signal Accumulation)
Flat Field	Applied (Lens)
Serial Number	UNKNOWN
Software Version	2.0.1
Application	Chemi
Excitation Source	No Illumination
Emission Filter	No Filter
Binning	2x2

Figure 2C.

FABPpm protein with reference gene GAPDH - Exposure time 7.6sec

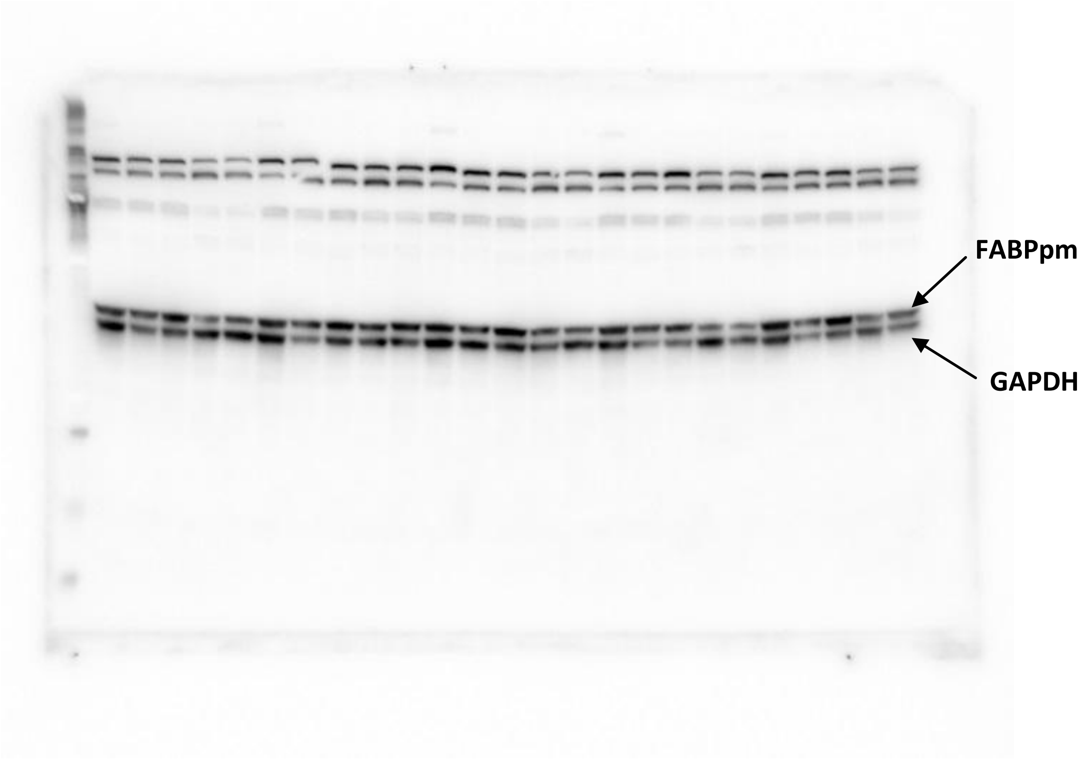

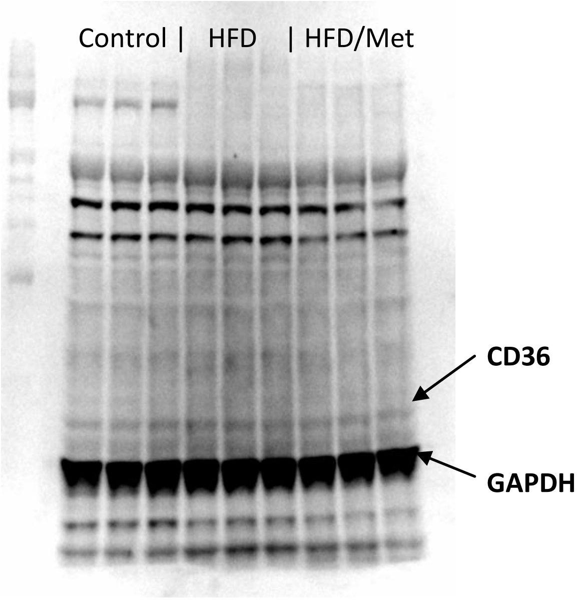

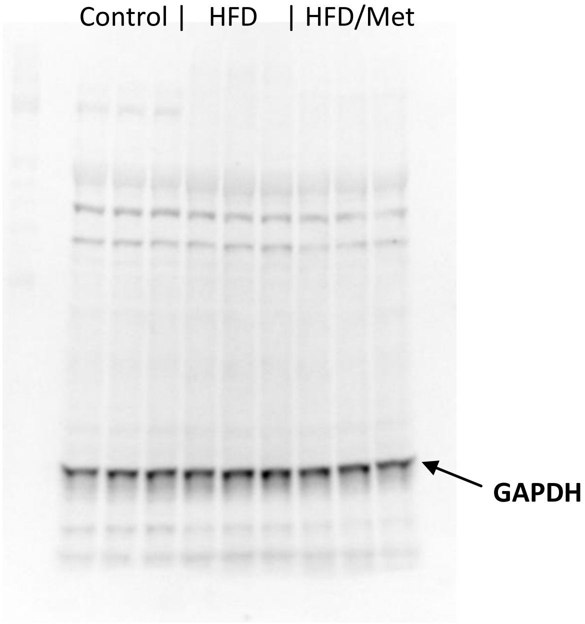

CD36 protein – Exposure time 59.0sec

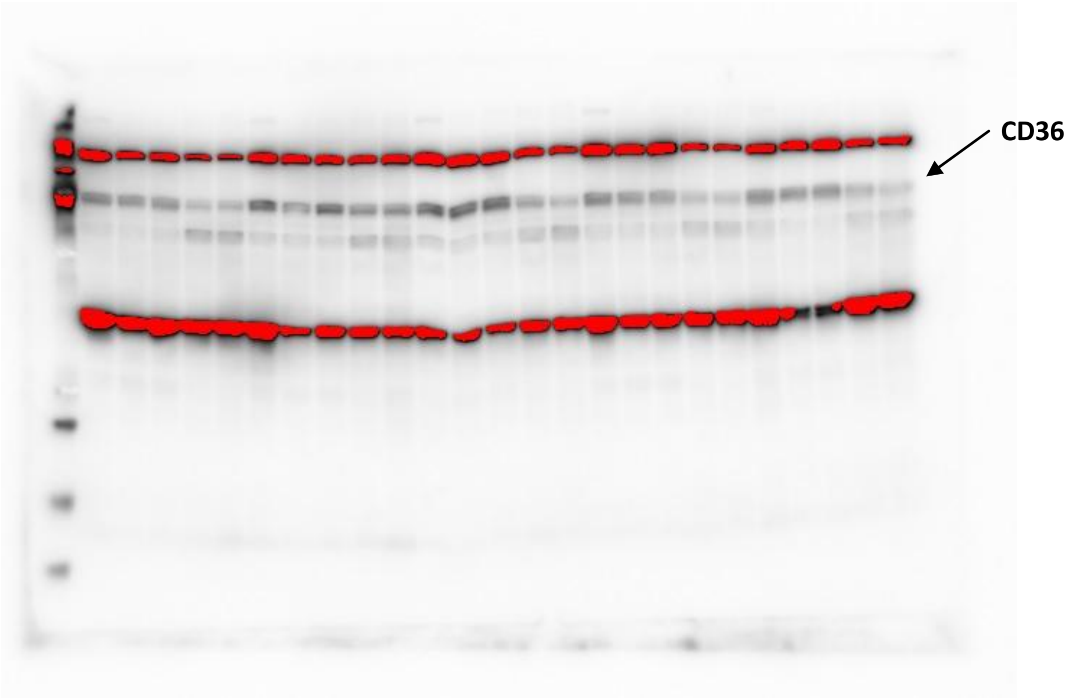

Reference gene GAPDH for CD36 protein - Exposure time 12.0sec

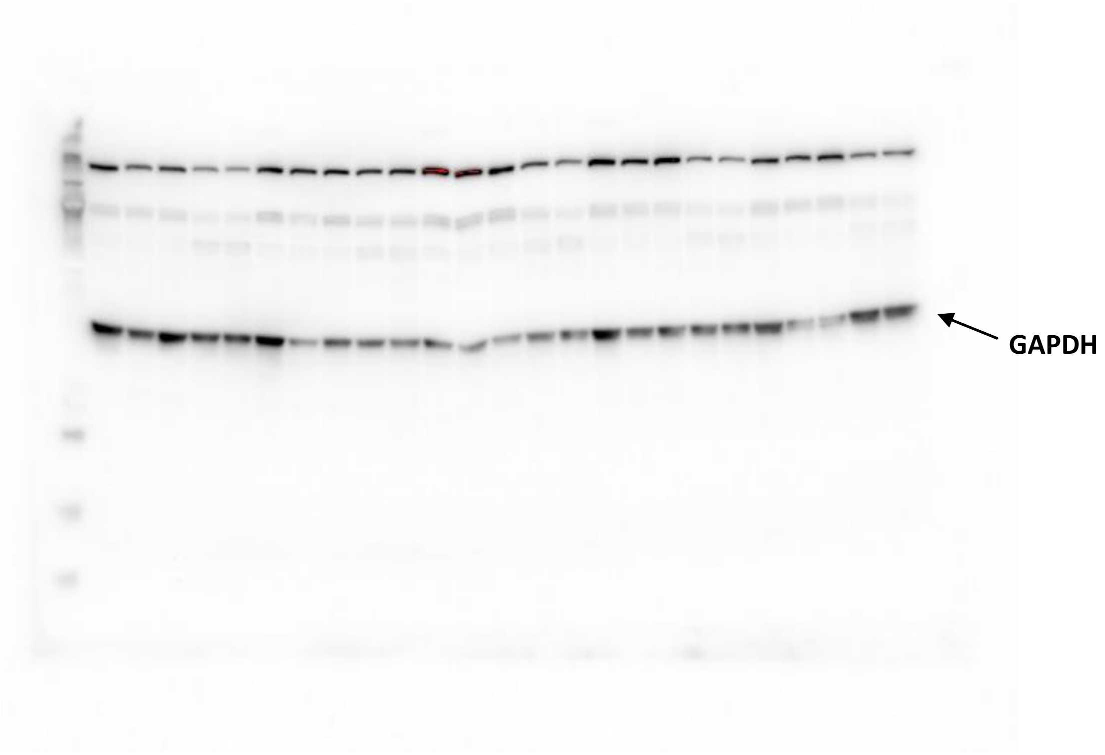

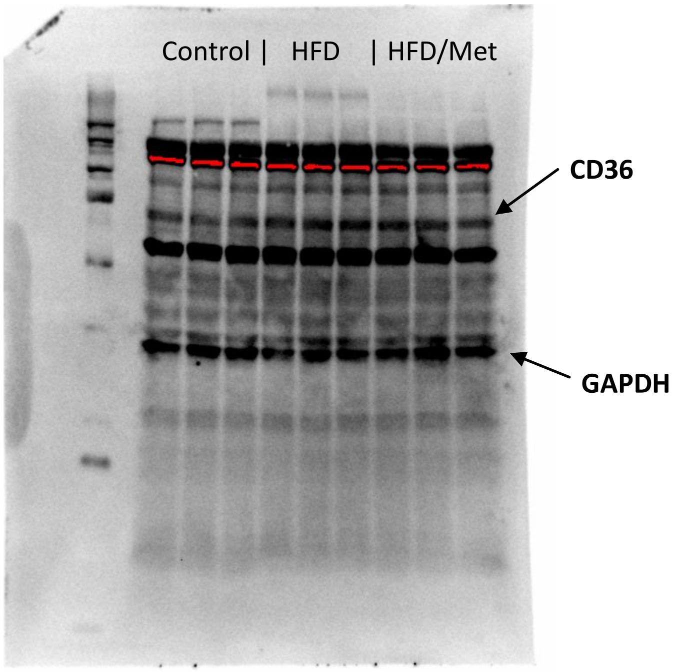

Control | HFD | HFD/Met
CD36
GAPDH

FATP2 protein with reference gene GAPDH – Exposure time 3.3sec

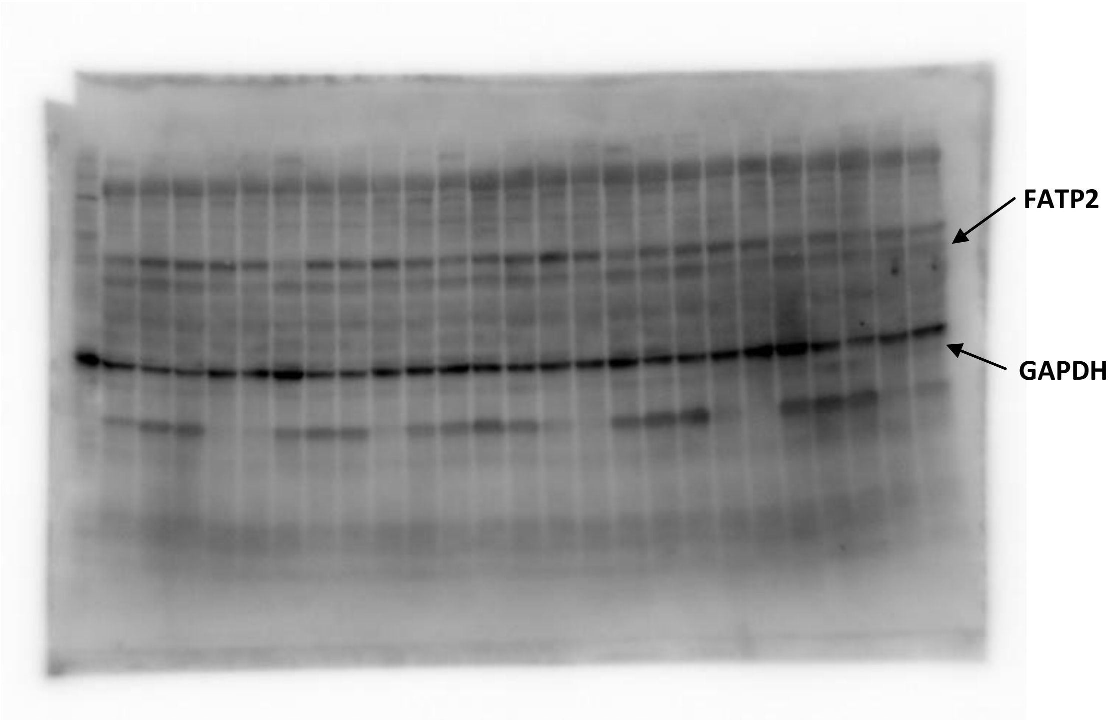

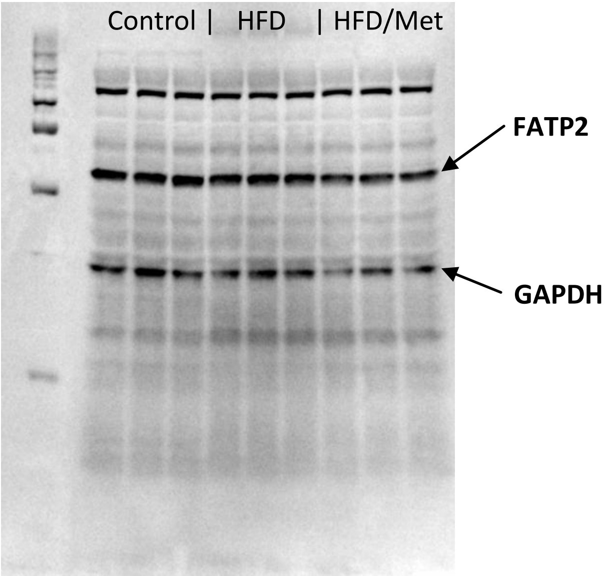

Figure 2D.

ACC protein with reference gene GAPDH - Exposure time 5.7sec

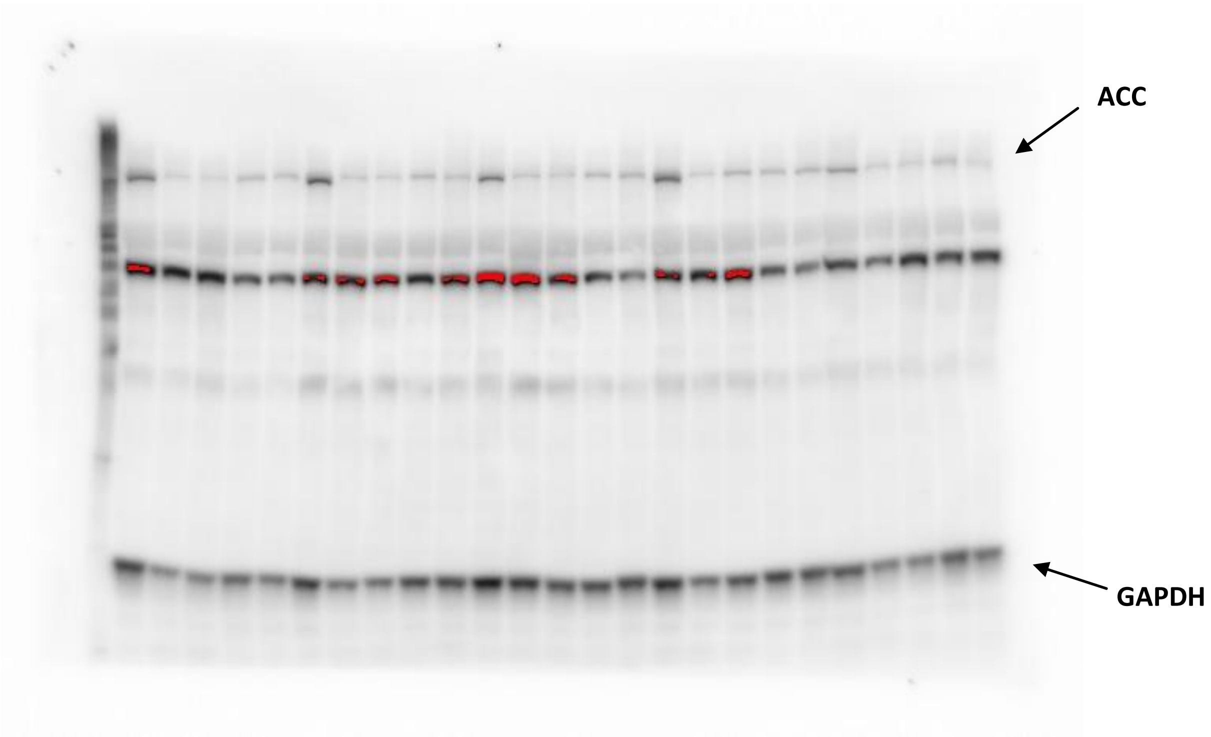

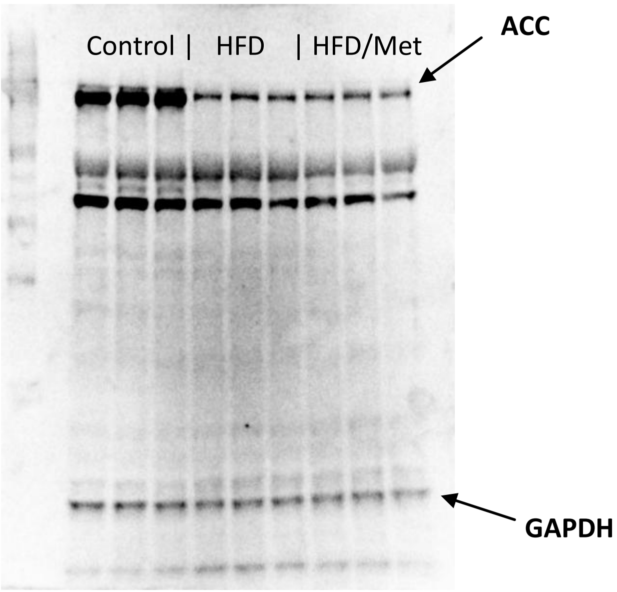

Figure 2E.

ACSVL1 protein with reference gene GAPDH – Exposure time 6.8sec

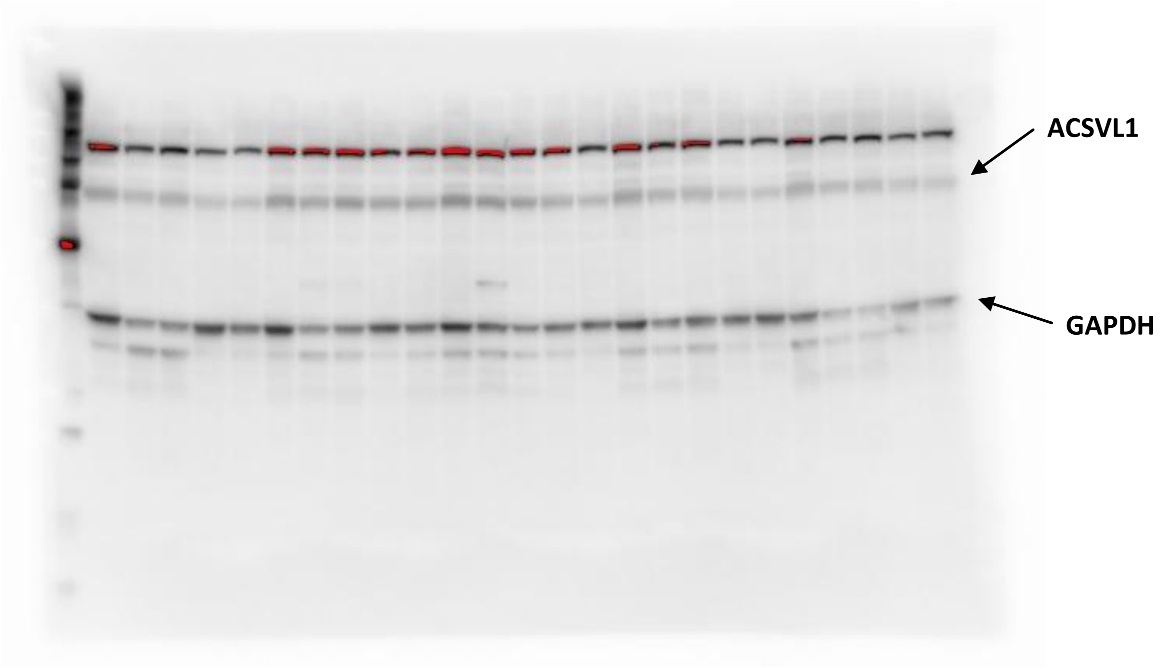

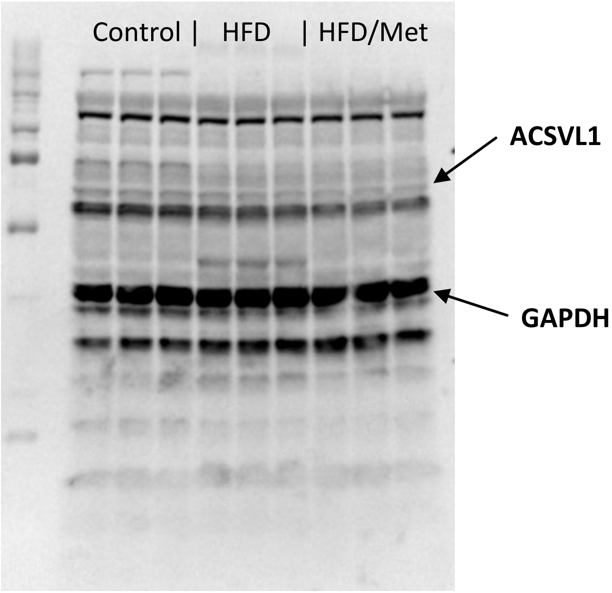

Figure 3E.

SPT protein with reference gene GAPDH – Exposure time 3.1sec

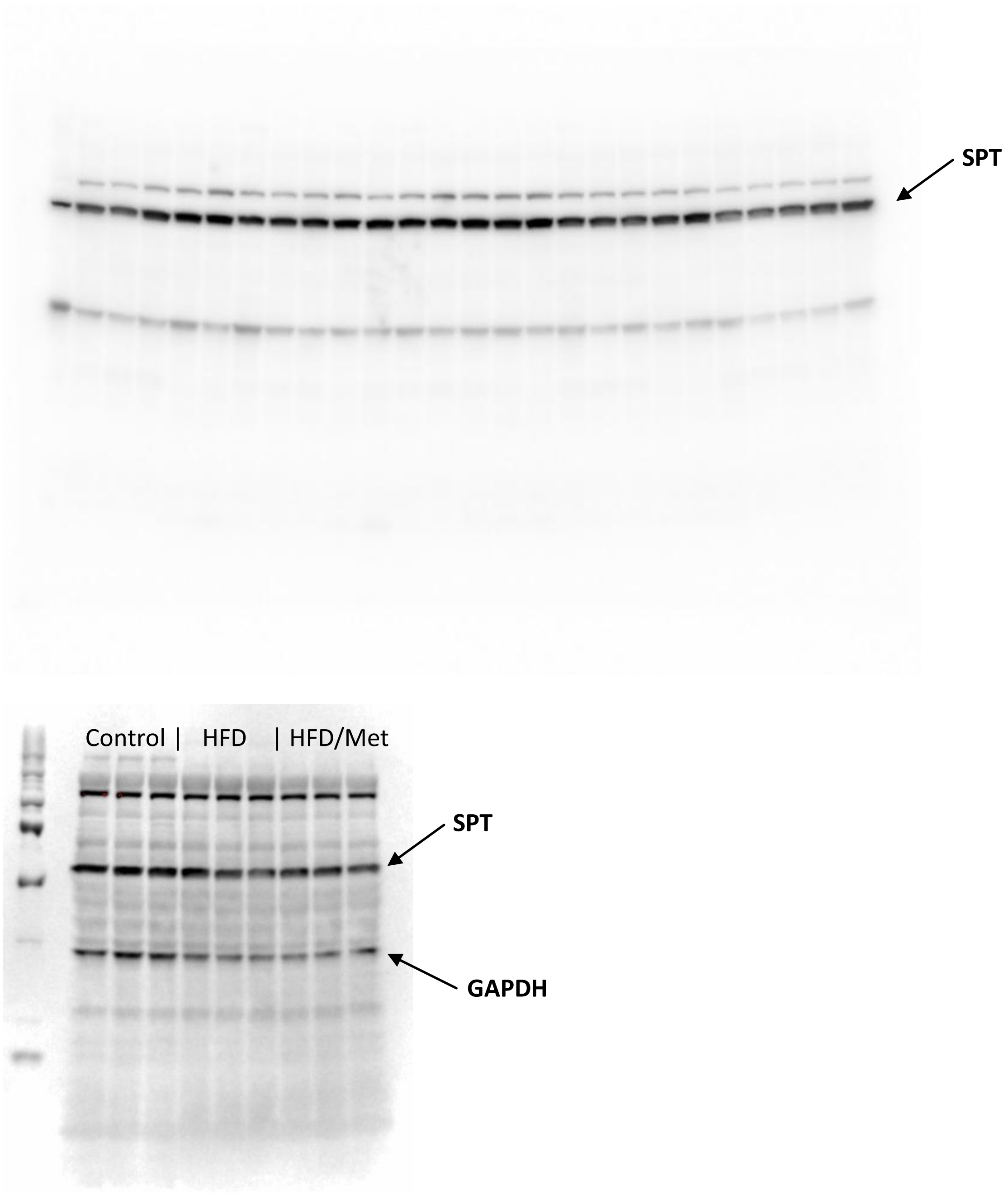

Figure 3F.

CerS4 protein – Exposure time 30.0sec

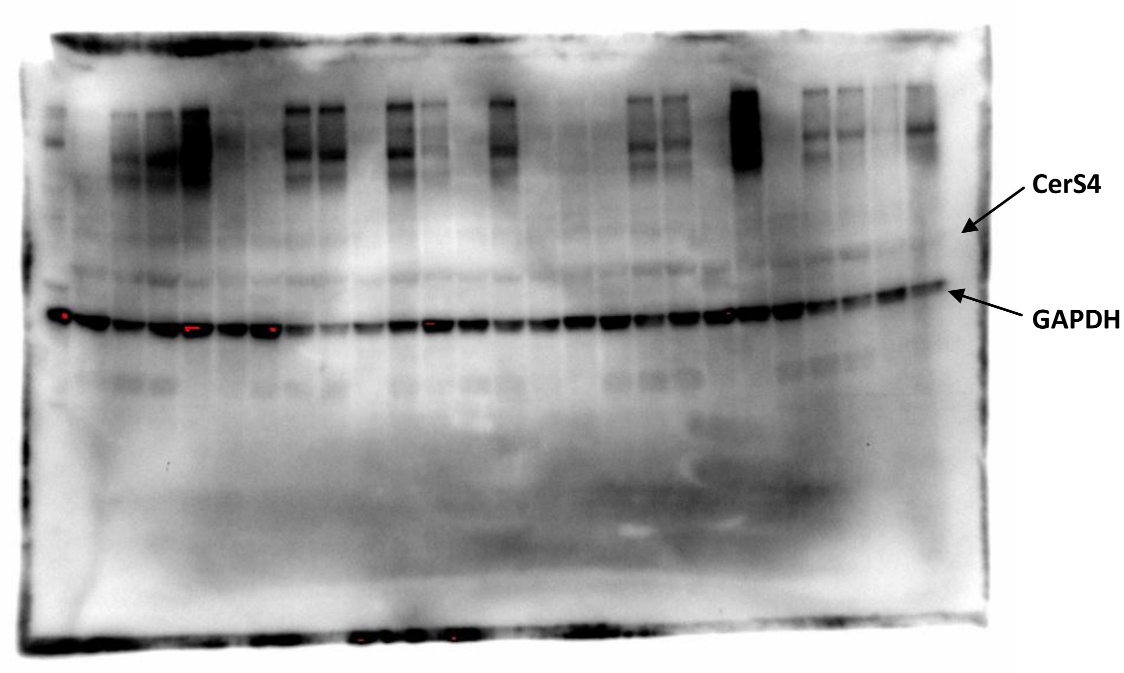

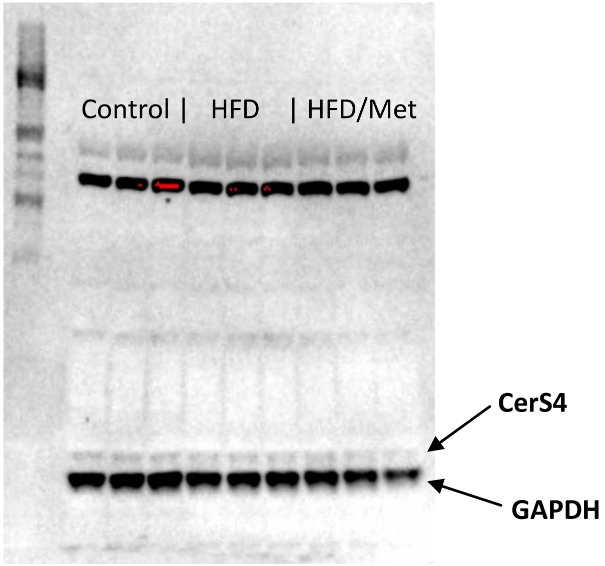

Reference gene GAPDH for CerS4 protein – Exposure time 27.0sec

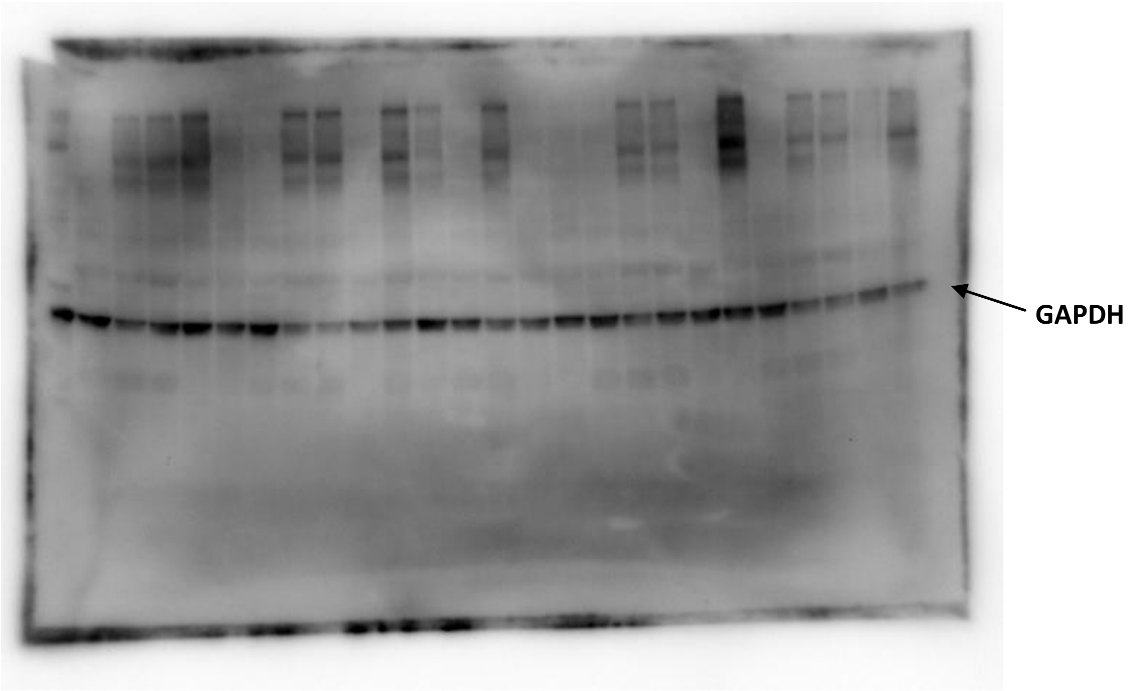

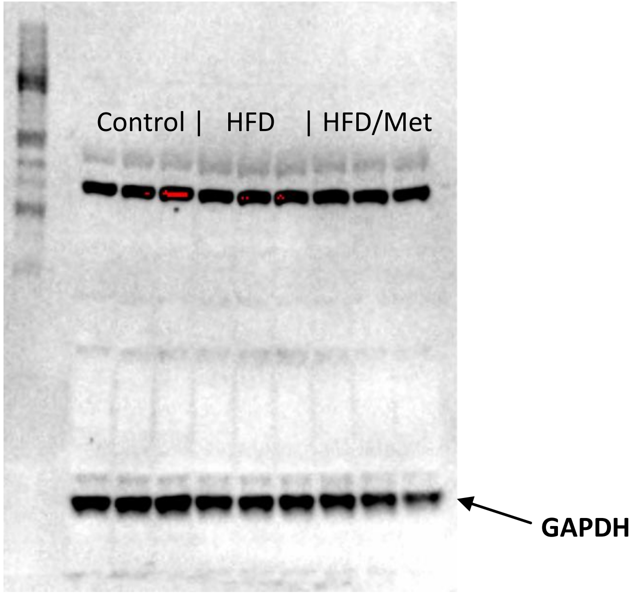

Figure 4C.

CPTI protein with reference gene GAPDH – Exposure time 13.0sec

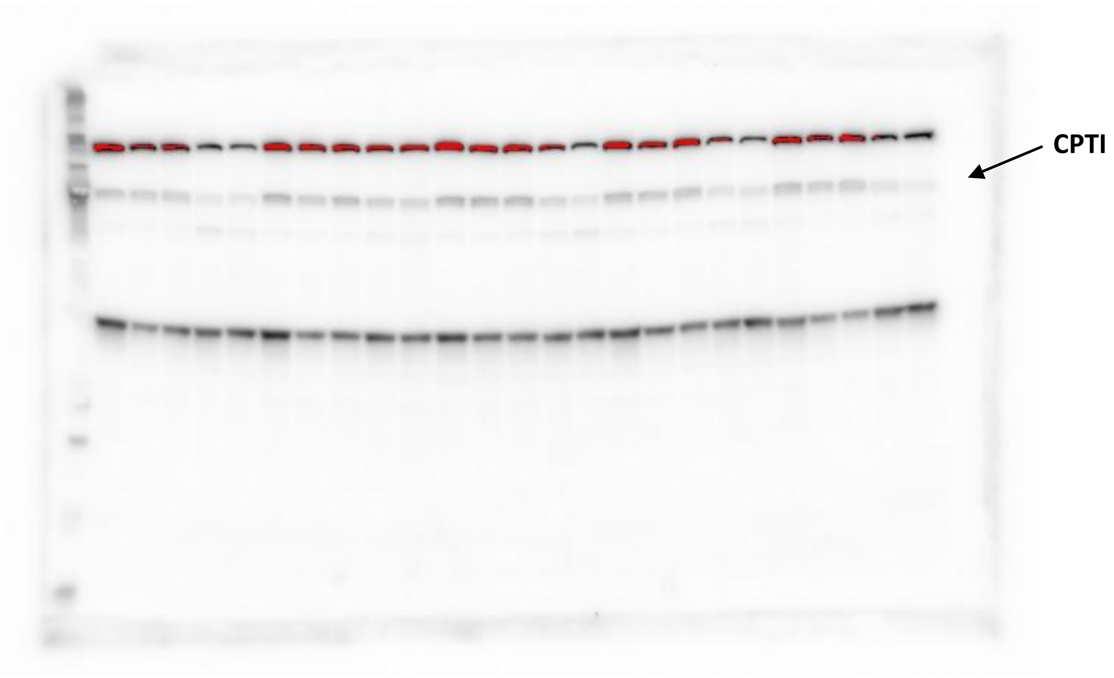

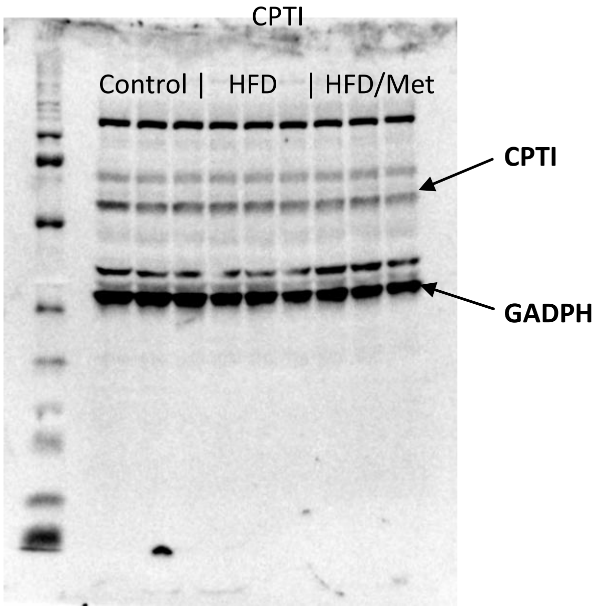

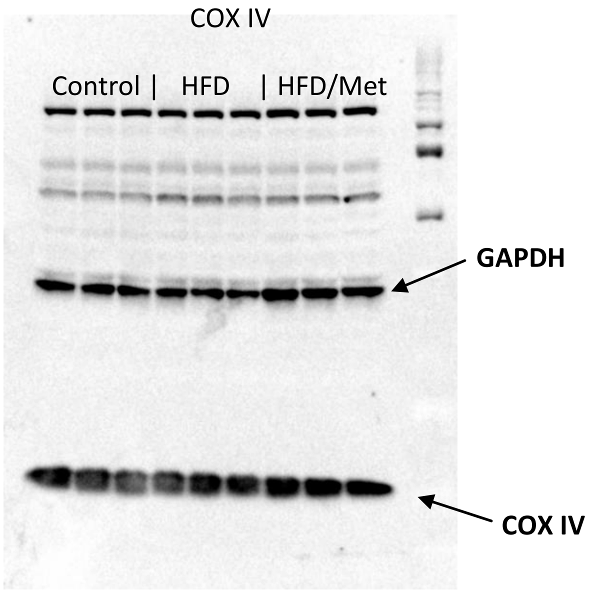
COX IV
Control | HFD | HFD/Met
GAPDH
COX IV

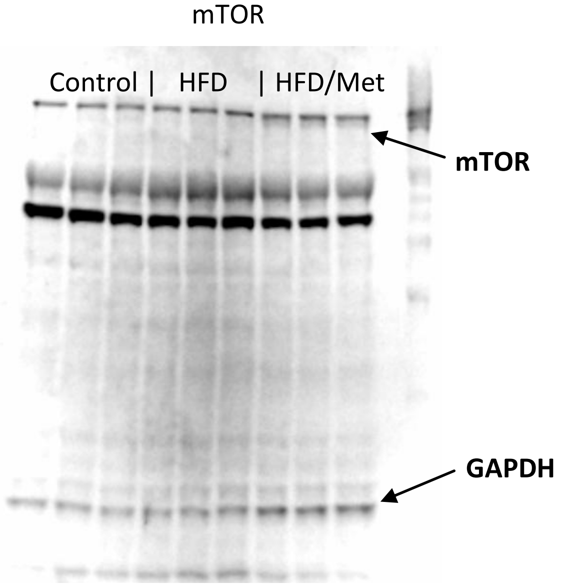
mTOR
Control | HFD | HFD/Met
mTOR
GAPDH

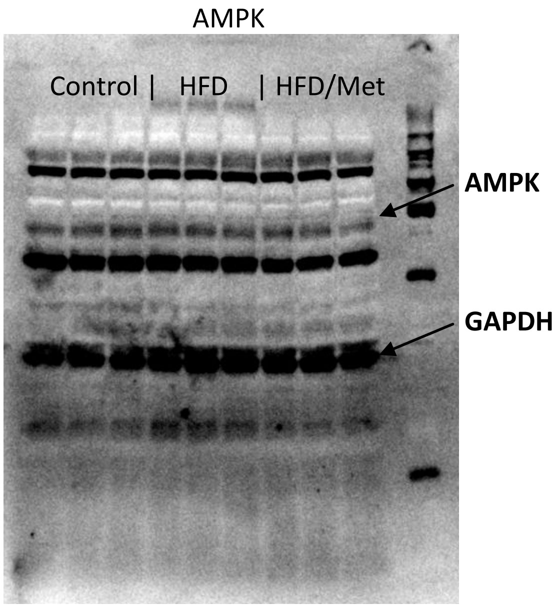
AMPK
Control | HFD | HFD/Met
AMPK
GAPDH

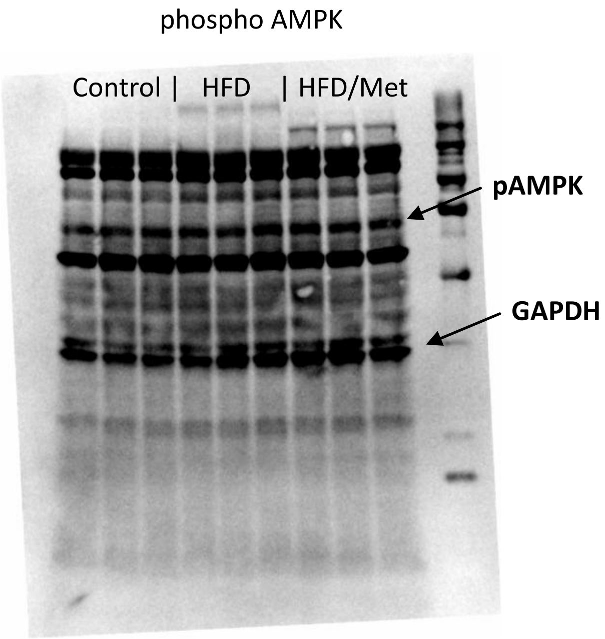
phospho AMPK
Control | HFD | HFD/Met
pAMPK
GAPDH

Figure 5. Particular proteins used to calculations of the ratio.

IRS1 protein with reference gene GAPDH – Exposure time 7.0sec

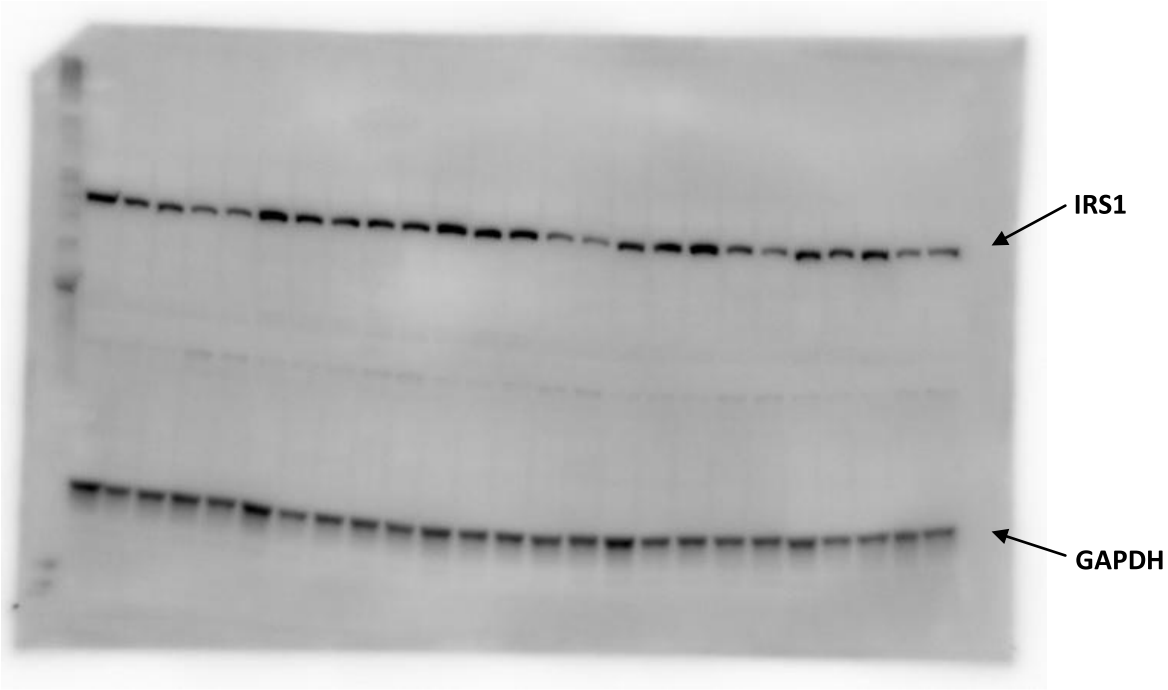

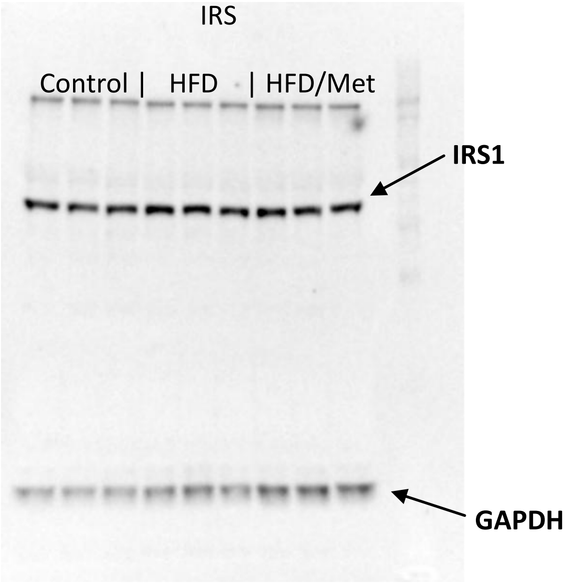

pIRS1(Tyr632) protein with reference gene GAPDH – Exposure time 3.3sec

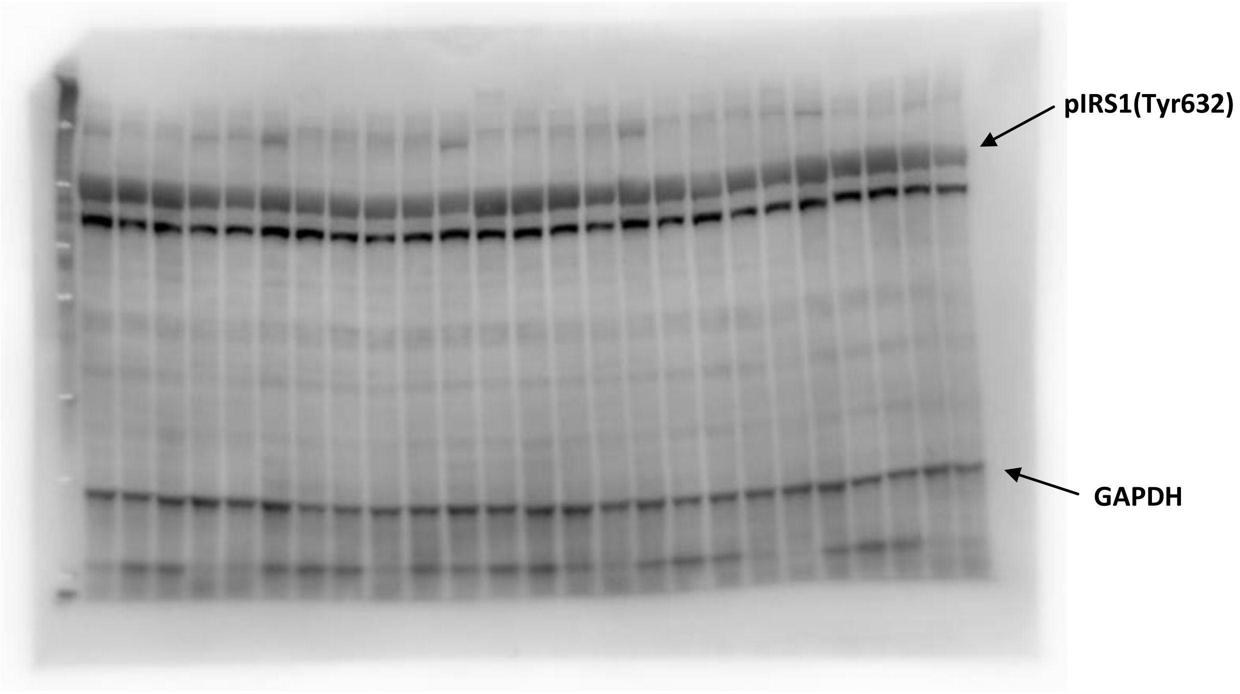

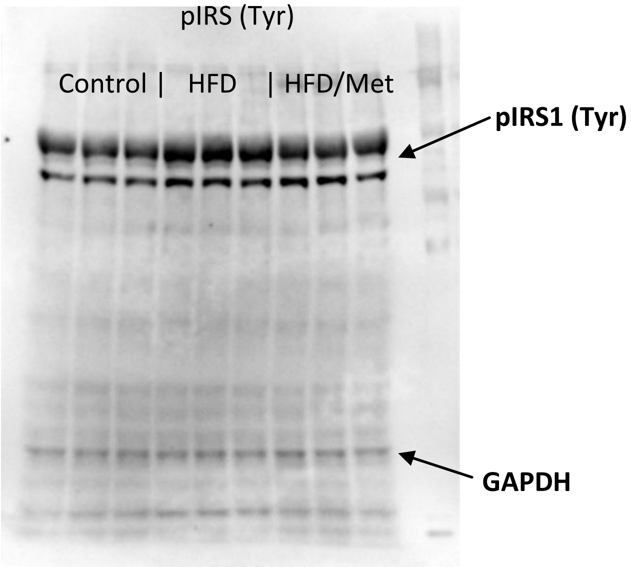

pIRS1(Ser270)protein with reference gene GAPDH – Exposure time 2.6sec

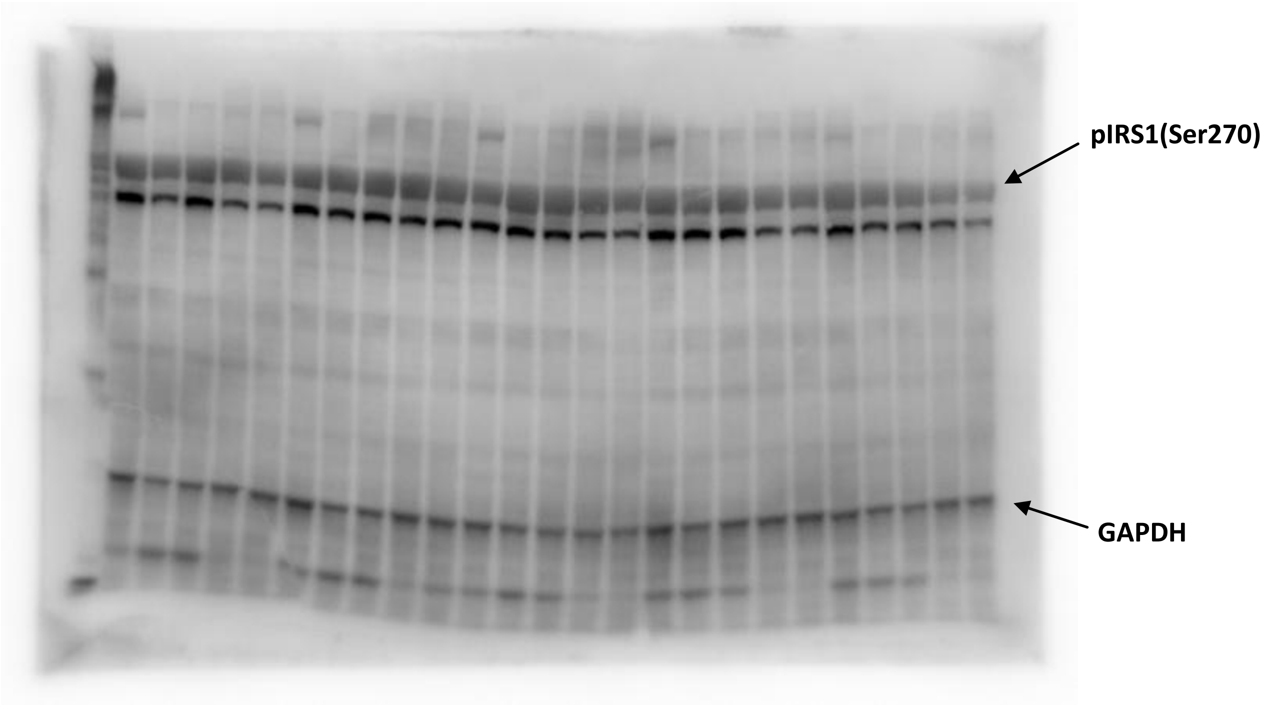

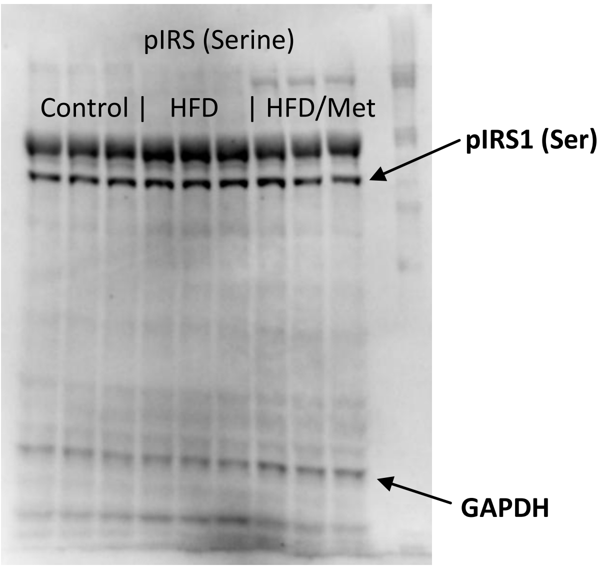

AKT protein with reference gene GAPDH – Exposure time 5.1sec

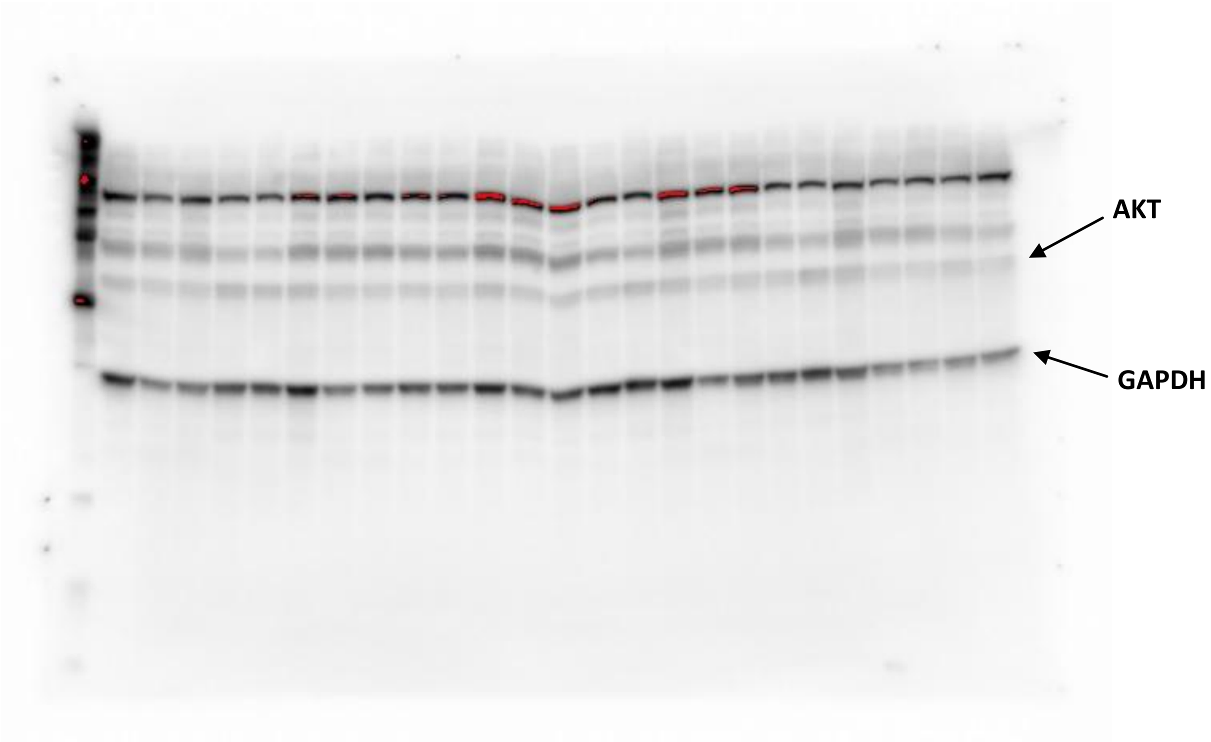

pAKT(Ser473) protein with reference gene GAPDH – Exposure time 6.4sec

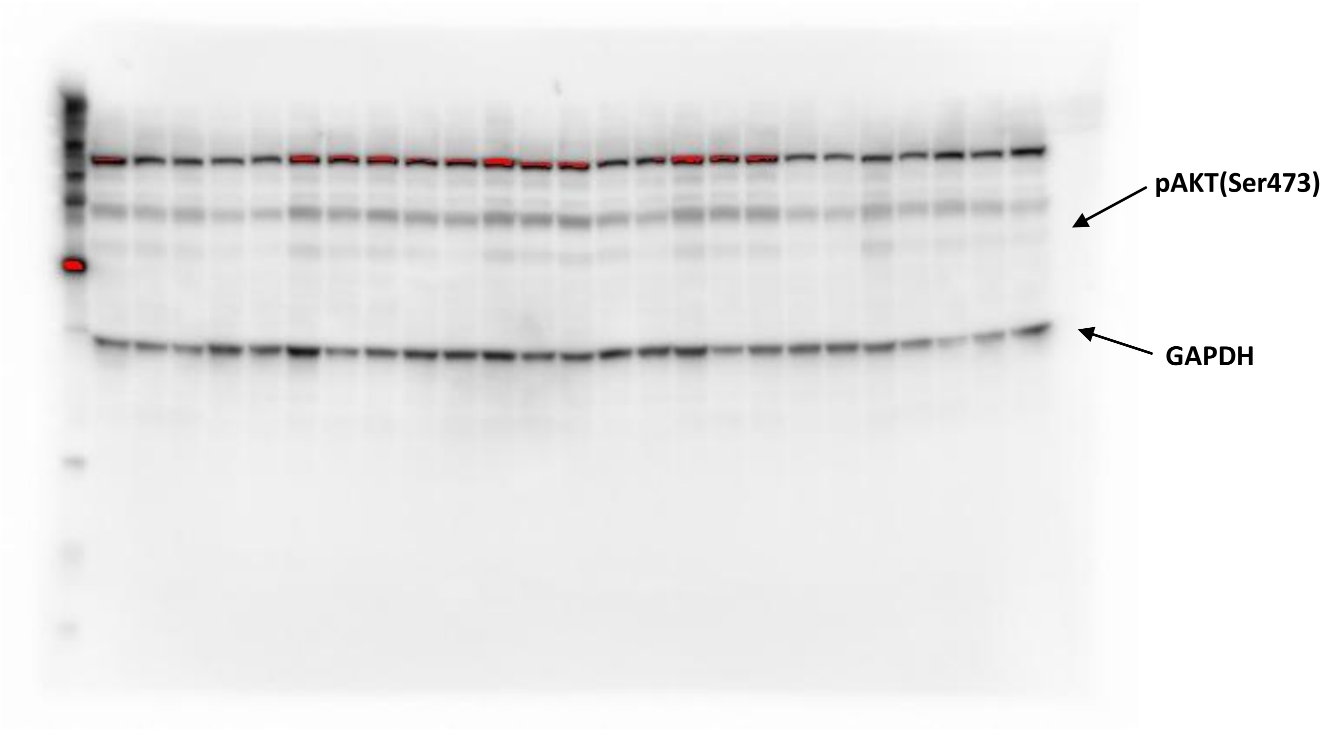

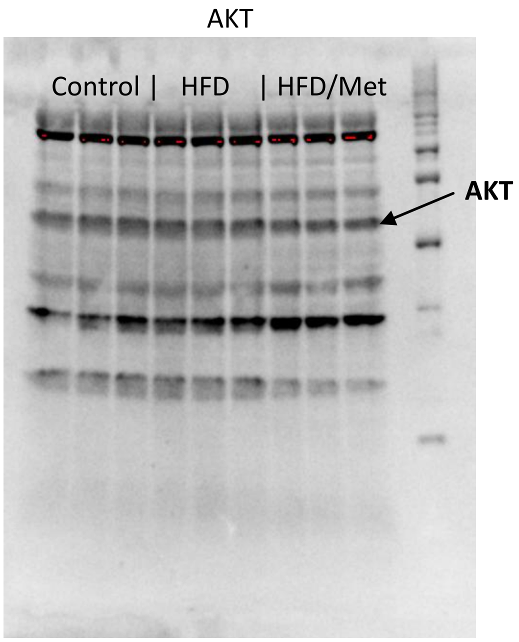
AKT
Control | HFD | HFD/Met
AKT

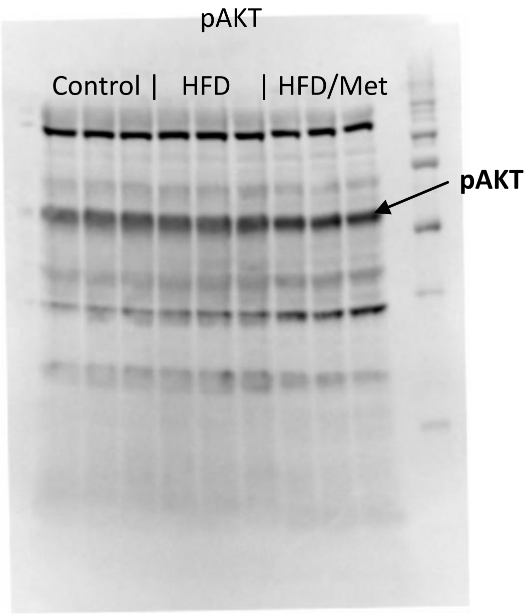
pAKT
Control | HFD | HFD/Met
pAKT

FoxO1 protein with reference gene GAPDH – Exposure time 9.7sec

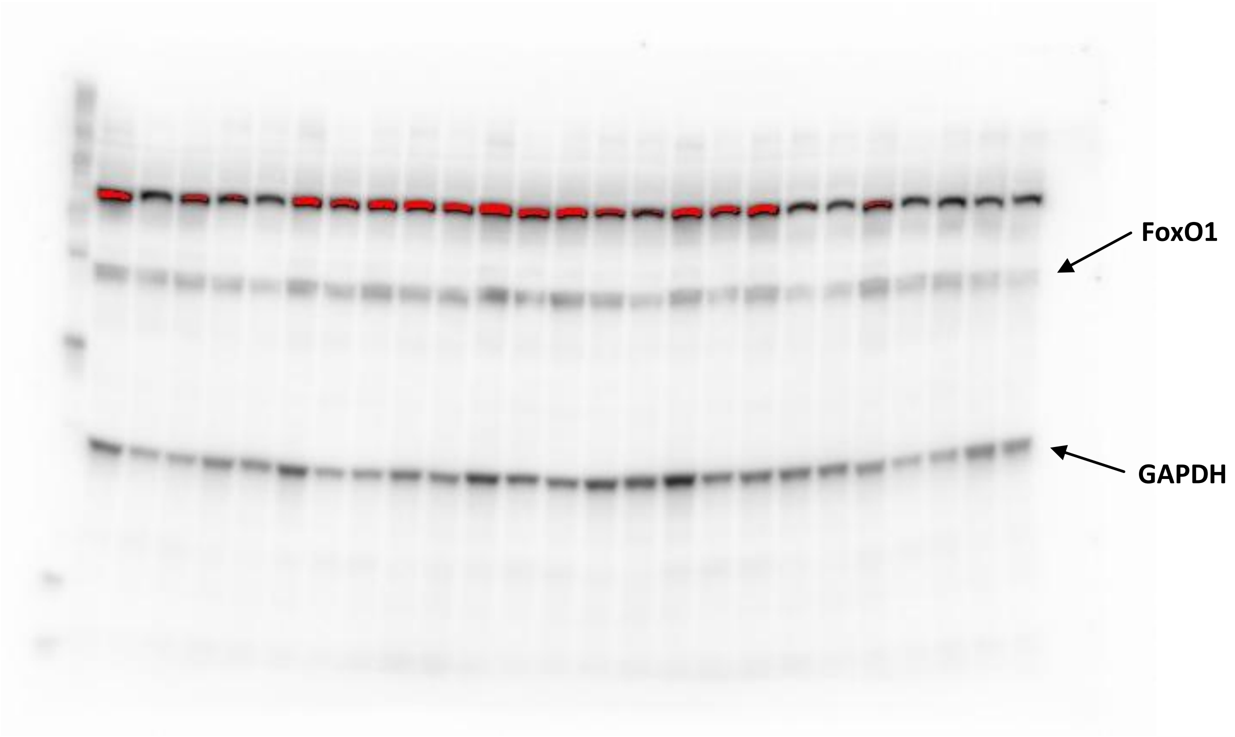

pFoxO1(Ser256) protein with reference gene GAPDH – Exposure time 9.8sec

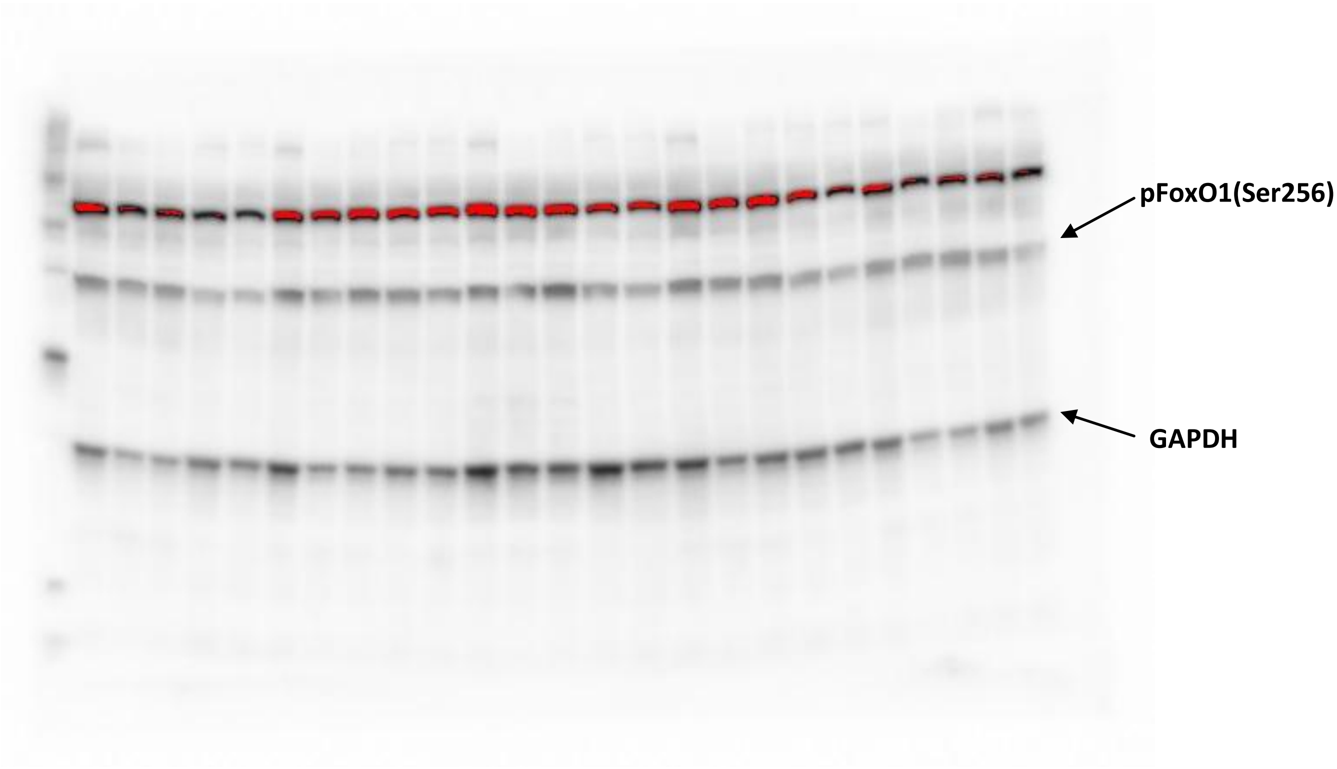

FoxO1

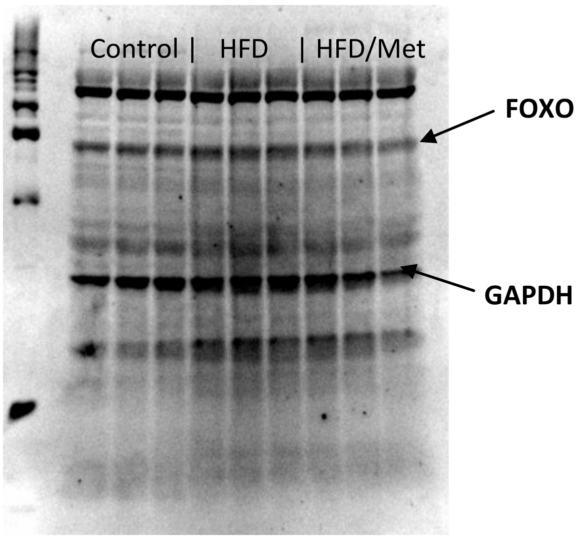

pFoxO1

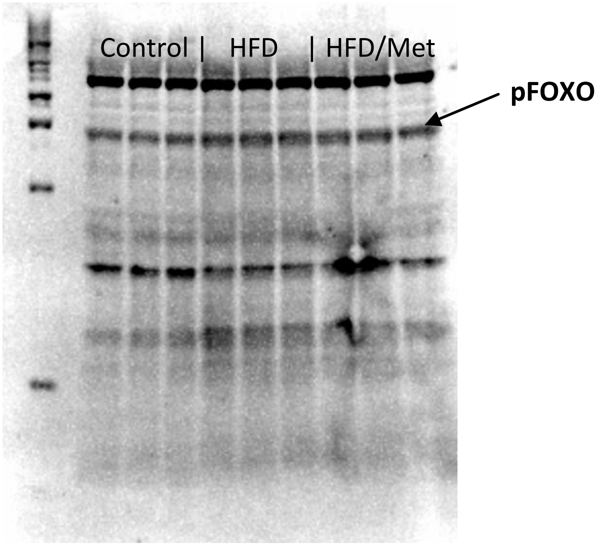

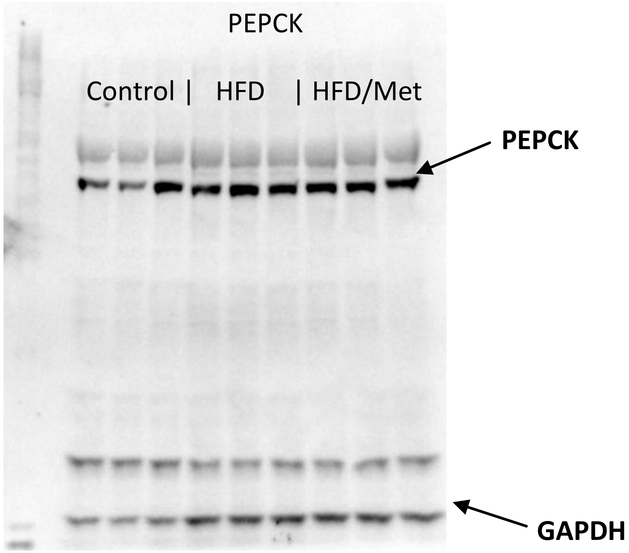

Supplement: Supplementary file 1 — Supplementary Information [file 41598_2018_25397_MOESM1_ESM.pdf]
